# Enzymes: principles and biotechnological applications

**DOI:** 10.1042/bse0590001

**Published:** 2015-10-26

**Authors:** Peter K. Robinson

**Affiliations:** College of Science and Technology, University of Central Lancashire, Preston PR1 2HE, U.K.

## Abstract

Enzymes are biological catalysts (also known as biocatalysts) that speed up biochemical reactions in living organisms, and which can be extracted from cells and then used to catalyse a wide range of commercially important processes. This chapter covers the basic principles of enzymology, such as classification, structure, kinetics and inhibition, and also provides an overview of industrial applications. In addition, techniques for the purification of enzymes are discussed.

## The nature and classification of enzymes

Enzymes are biological catalysts (also known as biocatalysts) that speed up biochemical reactions in living organisms. They can also be extracted from cells and then used to catalyse a wide range of commercially important processes. For example, they have important roles in the production of sweetening agents and the modification of antibiotics, they are used in washing powders and various cleaning products, and they play a key role in analytical devices and assays that have clinical, forensic and environmental applications. The word ‘enzyme’ was first used by the German physiologist Wilhelm Kühne in 1878, when he was describing the ability of yeast to produce alcohol from sugars, and it is derived from the Greek words *en* (meaning ‘within’) and *zume* (meaning ‘yeast’).

In the late nineteenth century and early twentieth century, significant advances were made in the extraction, characterization and commercial exploitation of many enzymes, but it was not until the 1920s that enzymes were crystallized, revealing that catalytic activity is associated with protein molecules. For the next 60 years or so it was believed that all enzymes were proteins, but in the 1980s it was found that some ribonucleic acid (RNA) molecules are also able to exert catalytic effects. These RNAs, which are called ribozymes, play an important role in gene expression. In the same decade, biochemists also developed the technology to generate antibodies that possess catalytic properties. These so-called ‘abzymes’ have significant potential both as novel industrial catalysts and in therapeutics. Notwithstanding these notable exceptions, much of classical enzymology, and the remainder of this essay, is focused on the proteins that possess catalytic activity.

As catalysts, enzymes are only required in very low concentrations, and they speed up reactions without themselves being consumed during the reaction. We usually describe enzymes as being capable of catalysing the conversion of substrate molecules into product molecules as follows:

$$\mathrm{Substrate}\overset{\mathrm{Enzyme}}{⇌}\mathrm{Product}$$

### Enzymes are potent catalysts

The enormous catalytic activity of enzymes can perhaps best be expressed by a constant, *k*_cat_, that is variously referred to as the turnover rate, turnover frequency or turnover number. This constant represents the number of substrate molecules that can be converted to product by a single enzyme molecule per unit time (usually per minute or per second). Examples of turnover rate values are listed in Table 1. For example, a single molecule of carbonic anhydrase can catalyse the conversion of over half a million molecules of its substrates, carbon dioxide (CO_2_) and water (H_2_O), into the product, bicarbonate (HCO_3_^−^), every second—a truly remarkable achievement.

**Table 1.** Turnover rate of some common enzymes showing wide variation.

Enzyme	Turnover rate (mole product s^−1^ mole enzyme^−1^)
Carbonic anhydrase	600 000
Catalase	93 000
β–galactosidase	200
Chymotrypsin	100
Tyrosinase	1

### Enzymes are specific catalysts

As well as being highly potent catalysts, enzymes also possess remarkable specificity in that they generally catalyse the conversion of only one type (or at most a range of similar types) of substrate molecule into product molecules.

Some enzymes demonstrate group specificity. For example, alkaline phosphatase (an enzyme that is commonly encountered in first-year laboratory sessions on enzyme kinetics) can remove a phosphate group from a variety of substrates.

Other enzymes demonstrate much higher specificity, which is described as absolute specificity. For example, glucose oxidase shows almost total specificity for its substrate, β-D-glucose, and virtually no activity with any other monosaccharides. As we shall see later, this specificity is of paramount importance in many analytical assays and devices (biosensors) that measure a specific substrate (e.g. glucose) in a complex mixture (e.g. a blood or urine sample).

### Enzyme names and classification

Enzymes typically have common names (often called ‘trivial names’) which refer to the reaction that they catalyse, with the suffix *-ase* (e.g. oxidase, dehydrogenase, carboxylase), although individual proteolytic enzymes generally have the suffix -*in* (e.g. trypsin, chymotrypsin, papain). Often the trivial name also indicates the substrate on which the enzyme acts (e.g. glucose oxidase, alcohol dehydrogenase, pyruvate decarboxylase). However, some trivial names (e.g. invertase, diastase, catalase) provide little information about the substrate, the product or the reaction involved.

Due to the growing complexity of and inconsistency in the naming of enzymes, the International Union of Biochemistry set up the Enzyme Commission to address this issue. The first Enzyme Commission Report was published in 1961, and provided a systematic approach to the naming of enzymes. The sixth edition, published in 1992, contained details of nearly 3 200 different enzymes, and supplements published annually have now extended this number to over 5 000.

Within this system, all enzymes are described by a four-part Enzyme Commission (EC) number. For example, the enzyme with the trivial name lactate dehydrogenase has the EC number 1.1.1.27, and is more correctly called l–lactate: NAD^+^ oxidoreductase.

The first part of the EC number refers to the reaction that the enzyme catalyses (Table 2). The remaining digits have different meanings according to the nature of the reaction identified by the first digit. For example, within the oxidoreductase category, the second digit denotes the hydrogen donor (Table 3) and the third digit denotes the hydrogen acceptor (Table 4).

**Table 2.** Enzyme Classification: Main classes of enzymes in EC system.

First EC digit	Enzyme class	Reaction type
1.	Oxidoreductases	Oxidation/reduction
2.	Transferases	Atom/group transfer (excluding other classes)
3.	Hydrolases	Hydrolysis
4.	Lyases	Group removal (excluding 3.)
5.	Isomerases	Isomerization
6.	Ligases	Joining of molecules linked to the breakage of a pyrophosphate bond

**Table 3.** Enzyme Classification: Secondary classes of oxidoreductase enzymes in EC system.

Oxidoreductases: second EC digit	Hydrogen or electron donor
1.	Alcohol (CHOH)
2.	Aldehyde or ketone (C═O)
3.	─CH─CH─
4.	Primary amine (CHNH_2_ or CHNH_3_^+^)
5.	Secondary amine (CHNH)
6.	NADH or NADPH (when another redox catalyst is the acceptor)

**Table 4.** Enzyme Classification: Tertiary classes of oxidoreductase enzymes in EC system.

Oxidoreductases: third EC digit	Hydrogen or electron acceptor
1.	NAD^+^ or NADP^+^
2.	Fe^3+^ (e.g. cytochromes)
3.	O_2_
4.	Other

Thus lactate dehydrogenase with the EC number 1.1.1.27 is an oxidoreductase (indicated by the first digit) with the alcohol group of the lactate molecule as the hydrogen donor (second digit) and NAD^+^ as the hydrogen acceptor (third digit), and is the 27th enzyme to be categorized within this group (fourth digit). 
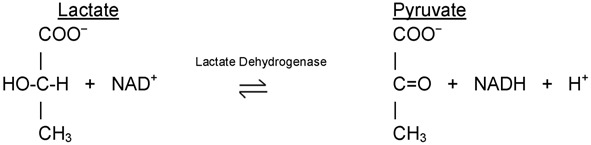


Fortunately, it is now very easy to find this information for any individual enzyme using the Enzyme Nomenclature Database (available at http://enzyme.expasy.org).

## Enzyme structure and substrate binding

**Figure 1.**
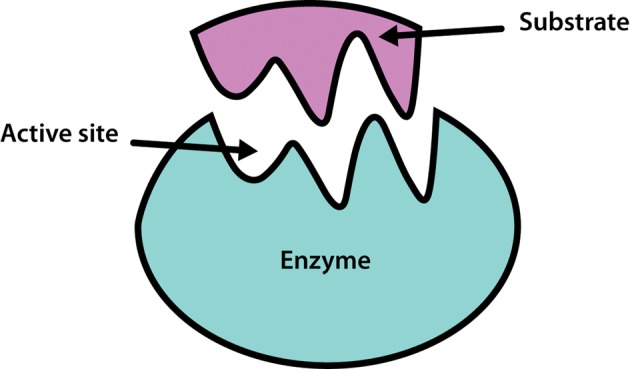
Representation of substrate binding to the active site of an enzyme molecule.

Amino acid-based enzymes are globular proteins that range in size from less than 100 to more than 2 000 amino acid residues. These amino acids can be arranged as one or more polypeptide chains that are folded and bent to form a specific three-dimensional structure, incorporating a small area known as the active site (Figure 1), where the substrate actually binds. The active site may well involve only a small number (less than 10) of the constituent amino acids.

It is the shape and charge properties of the active site that enable it to bind to a single type of substrate molecule, so that the enzyme is able to demonstrate considerable specificity in its catalytic activity.

The hypothesis that enzyme specificity results from the complementary nature of the substrate and its active site was first proposed by the German chemist Emil Fischer in 1894, and became known as Fischer's ‘lock and key hypothesis’, whereby only a key of the correct size and shape (the substrate) fits into the keyhole (the active site) of the lock (the enzyme). It is astounding that this theory was proposed at a time when it was not even established that enzymes were proteins. As more was learned about enzyme structure through techniques such as X-ray crystallography, it became clear that enzymes are not rigid structures, but are in fact quite flexible in shape. In the light of this finding, in 1958 Daniel Koshland extended Fischer's ideas and presented the ‘induced-fit model’ of substrate and enzyme binding, in which the enzyme molecule changes its shape slightly to accommodate the binding of the substrate. The analogy that is commonly used is the ‘hand-in-glove model’, where the hand and glove are broadly complementary in shape, but the glove is moulded around the hand as it is inserted in order to provide a perfect match.

Since it is the active site alone that binds to the substrate, it is logical to ask what is the role of the rest of the protein molecule. The simple answer is that it acts to stabilize the active site and provide an appropriate environment for interaction of the site with the substrate molecule. Therefore the active site cannot be separated out from the rest of the protein without loss of catalytic activity, although laboratory-based directed (or forced) evolution studies have shown that it is sometimes possible to generate smaller enzymes that do retain activity.

It should be noted that although a large number of enzymes consist solely of protein, many also contain a non-protein component, known as a cofactor, that is necessary for the enzyme's catalytic activity. A cofactor may be another organic molecule, in which case it is called a coenzyme, or it may be an inorganic molecule, typically a metal ion such as iron, manganese, cobalt, copper or zinc. A coenzyme that binds tightly and permanently to the protein is generally referred to as the prosthetic group of the enzyme.

**Figure 2.**
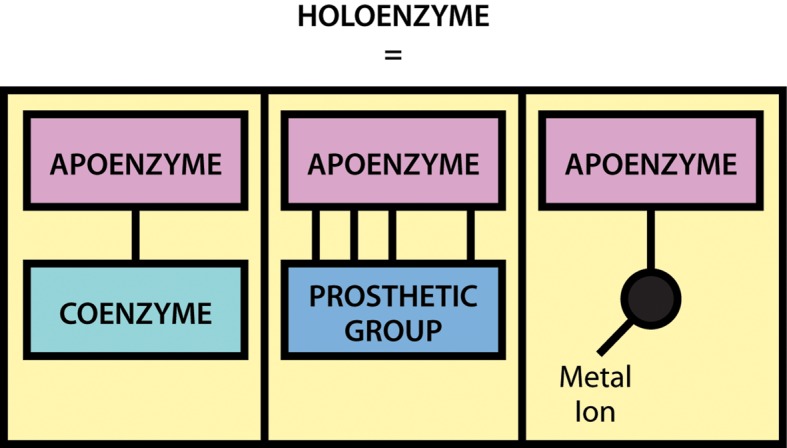
The components of a holoenzyme.

When an enzyme requires a cofactor for its activity, the inactive protein component is generally referred to as an apoenzyme, and the apoenzyme plus the cofactor (i.e. the active enzyme) is called a holoenzyme (Figure 2).

The need for minerals and vitamins in the human diet is partly attributable to their roles within metabolism as cofactors and coenzymes.

### Enzymes and reaction equilibrium

How do enzymes work? The broad answer to this question is that they do not alter the equilibrium (i.e. the thermodynamics) of a reaction. This is because enzymes do not fundamentally change the structure and energetics of the products and reagents, but rather they simply allow the reaction equilibrium to be attained more rapidly. Let us therefore begin by clarifying the concept of chemical equilibrium.

In many cases the equilibrium of a reaction is far ‘to the right’—that is, virtually all of the substrate (S) is converted into product (P). For this reason, reactions are often written as follows:

S→P

This is a simplification, as in all cases it is more correct to write this reaction as follows:

S⇌P

This indicates the presence of an equilibrium. To understand this concept it is perhaps most helpful to look at a reaction where the equilibrium point is quite central.

For example:

$$\mathrm{Glucose}\overset{\text{Glucose isomerase}}{⇌}\mathrm{Fructose}$$

In this reaction, if we start with a solution of 1 mol l^−1^ glucose and add the enzyme, then upon completion we will have a mixture of approximately 0.5 mol l^−1^ glucose and 0.5 mol l^−1^ fructose. This is the equilibrium point of this particular reaction, and although it may only take a couple of seconds to reach this end point with the enzyme present, we would in fact come to the same point if we put glucose into solution and waited many months for the reaction to occur in the absence of the enzyme. Interestingly, we could also have started this reaction with a 1 mol l^−1^ fructose solution, and it would have proceeded in the opposite direction until the same equilibrium point had been reached.

The equilibrium point for this reaction is expressed by the equilibrium constant *K*_eq_ as follows:

$$K_{\mathrm{eq}}=\frac{\text{Substrate concentration at end point}}{\text{Substrate concentration at end point}}=\frac{0.5}{0.5}=1$$

Thus for a reaction with central equilibrium, *K*_eq_ = 1, for an equilibrium ‘to the right’ *K*_eq_ is >1, and for an equilibrium ‘to the left’ *K*_eq_ is <1.

Therefore if a reaction has a *K*_eq_ value of 10^6^, the equilibrium is very far to the right and can be simplified by denoting it as a single arrow. We may often describe this type of reaction as ‘going to completion’. Conversely, if a reaction has a *K*_eq_ value of 10^−6^, the equilibrium is very far to the left, and for all practical purposes it would not really be considered to proceed at all.

It should be noted that although the concentration of reactants has no effect on the equilibrium point, environmental factors such as pH and temperature can and do affect the position of the equilibrium.

It should also be noted that any biochemical reaction which occurs *in vivo* in a living system does not occur in isolation, but as part of a metabolic pathway, which makes it more difficult to conceptualize the relationship between reactants and reactions. *In vivo* reactions are not allowed to proceed to their equilibrium position. If they did, the reaction would essentially stop (i.e. the forward and reverse reactions would balance each other), and there would be no net flux through the pathway. However, in many complex biochemical pathways some of the individual reaction steps are close to equilibrium, whereas others are far from equilibrium, the latter (catalysed by regulatory enzymes) having the greatest capacity to control the overall flux of materials through the pathway.

### Enzymes form complexes with their substrates

We often describe an enzyme-catalysed reaction as proceeding through three stages as follows:

E+S→EScomplex→E+P

The ES complex represents a position where the substrate (S) is bound to the enzyme (E) such that the reaction (whatever it might be) is made more favourable. As soon as the reaction has occurred, the product molecule (P) dissociates from the enzyme, which is then free to bind to another substrate molecule. At some point during this process the substrate is converted into an intermediate form (often called the transition state) and then into the product.

The exact mechanism whereby the enzyme acts to increase the rate of the reaction differs from one system to another. However, the general principle is that by binding of the substrate to the enzyme, the reaction involving the substrate is made more favourable by lowering the activation energy of the reaction.

In terms of energetics, reactions can be either exergonic (releasing energy) or endergonic (consuming energy). However, even in an exergonic reaction a small amount of energy, termed the activation energy, is needed to give the reaction a ‘kick start.’ A good analogy is that of a match, the head of which contains a mixture of energy-rich chemicals (phosphorus sesquisulfide and potassium chlorate). When a match burns it releases substantial amounts of light and heat energy (exergonically reacting with O_2_ in the air). However, and perhaps fortunately, a match will not spontaneously ignite, but rather a small input of energy in the form of heat generated through friction (i.e. striking of the match) is needed to initiate the reaction. Of course once the match has been struck the amount of energy released is considerable, and greatly exceeds the small energy input during the striking process.

**Figure 3.**
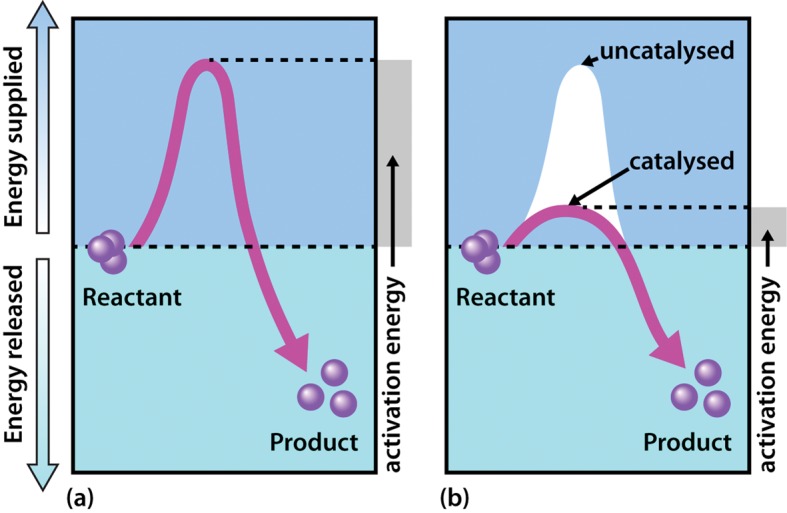
Effect of an enzyme on reducing the activation energy required to start a reaction where (a) is uncatalysed and (b) is enzyme-catalysed reaction.

As shown in Figure 3, enzymes are considered to lower the activation energy of a system by making it energetically easier for the transition state to form. In the presence of an enzyme catalyst, the formation of the transition state is energetically more favourable (i.e. it requires less energy for the ‘kick start’), thereby accelerating the rate at which the reaction will proceed, but not fundamentally changing the energy levels of either the reactant or the product.

## Properties and mechanisms of enzyme action

### Enzyme kinetics

Enzyme kinetics is the study of factors that determine the speed of enzyme-catalysed reactions. It utilizes some mathematical equations that can be confusing to students when they first encounter them. However, the theory of kinetics is both logical and simple, and it is essential to develop an understanding of this subject in order to be able to appreciate the role of enzymes both in metabolism and in biotechnology.

Assays (measurements) of enzyme activity can be performed in either a discontinuous or continuous fashion. Discontinuous methods involve mixing the substrate and enzyme together and measuring the product formed after a set period of time, so these methods are generally easy and quick to perform. In general we would use such discontinuous assays when we know little about the system (and are making preliminary investigations), or alternatively when we know a great deal about the system and are certain that the time interval we are choosing is appropriate.

In continuous enzyme assays we would generally study the rate of an enzyme-catalysed reaction by mixing the enzyme with the substrate and continuously measuring the appearance of product over time. Of course we could equally well measure the rate of the reaction by measuring the disappearance of substrate over time. Apart from the actual direction (one increasing and one decreasing), the two values would be identical. In enzyme kinetics experiments, for convenience we very often use an artificial substrate called a chromogen that yields a brightly coloured product, making the reaction easy to follow using a colorimeter or a spectrophotometer. However, we could in fact use any available analytical equipment that has the capacity to measure the concentration of either the product or the substrate. 
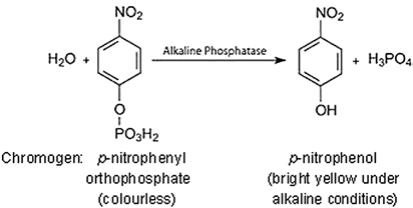


In almost all cases we would also add a buffer solution to the mixture. As we shall see, enzyme activity is strongly influenced by pH, so it is important to set the pH at a specific value and keep it constant throughout the experiment.

**Figure 4.**
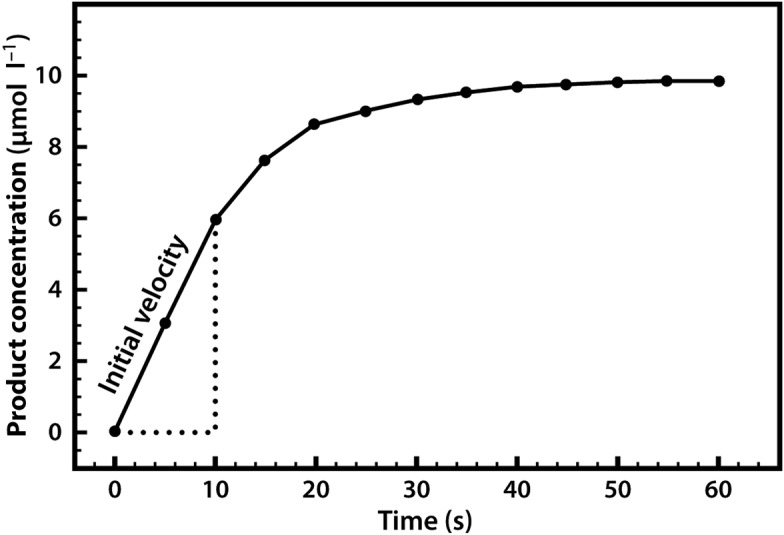
Formation of product in an enzyme-catalysed reaction, plotted against time.

Our first enzyme kinetics experiment may therefore involve mixing a substrate solution (chromogen) with a buffer solution and adding the enzyme. This mixture would then be placed in a spectrophotometer and the appearance of the coloured product would be measured. This would enable us to follow a rapid reaction which, after a few seconds or minutes, might start to slow down, as shown in Figure 4.

A common reason for this slowing down of the speed (rate) of the reaction is that the substrate within the mixture is being used up and thus becoming limiting. Alternatively, it may be that the enzyme is unstable and is denaturing over the course of the experiment, or it could be that the pH of the mixture is changing, as many reactions either consume or release protons. For these reasons, when we are asked to specify the rate of a reaction we do so early on, as soon as the enzyme has been added, and when none of the above-mentioned limitations apply. We refer to this initial rapid rate as the initial velocity (*v*_0_). Measurement of the reaction rate at this early stage is also quite straightforward, as the rate is effectively linear, so we can simply draw a straight line and measure the gradient (by dividing the concentration change by the time interval) in order to evaluate the reaction rate over this period.

We may now perform a range of similar enzyme assays to evaluate how the initial velocity changes when the substrate or enzyme concentration is altered, or when the pH is changed. These studies will help us to characterize the properties of the enzyme under study.

**Figure 5.**
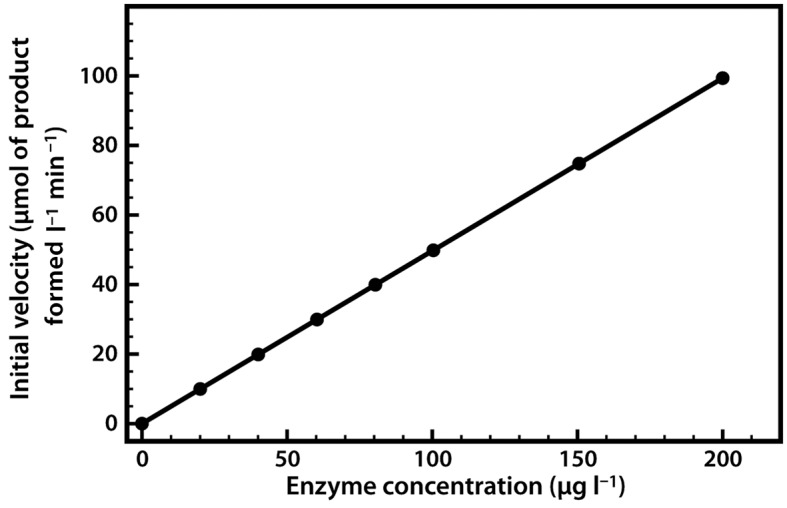
Relationship between enzyme concentration and the rate of an enzyme-catalysed reaction.

The relationship between enzyme concentration and the rate of the reaction is usually a simple one. If we repeat the experiment just described, but add 10% more enzyme, the reaction will be 10% faster, and if we double the enzyme concentration the reaction will proceed twice as fast. Thus there is a simple linear relationship between the reaction rate and the amount of enzyme available to catalyse the reaction (Figure 5).

This relationship applies both to enzymes *in vivo* and to those used in biotechnological applications, where regulation of the amount of enzyme present may control reaction rates.

**Figure 6.**
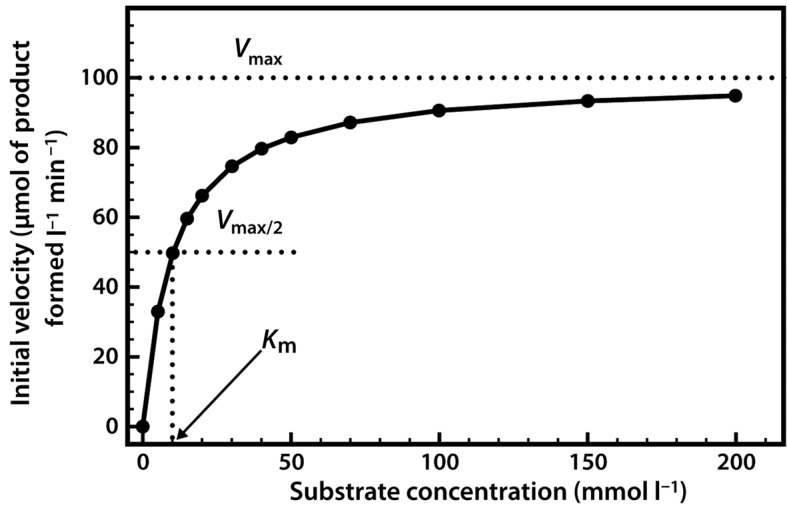
Relationship between substrate concentration and the rate of an enzyme-catalysed reaction.

When we perform a series of enzyme assays using the same enzyme concentration, but with a range of different substrate concentrations, a slightly more complex relationship emerges, as shown in Figure 6. Initially, when the substrate concentration is increased, the rate of reaction increases considerably. However, as the substrate concentration is increased further the effects on the reaction rate start to decline, until a stage is reached where increasing the substrate concentration has little further effect on the reaction rate. At this point the enzyme is considered to be coming close to saturation with substrate, and demonstrating its maximal velocity (*V*_max_). Note that this maximal velocity is in fact a theoretical limit that will not be truly achieved in any experiment, although we might come very close to it.

The relationship described here is a fairly common one, which a mathematician would immediately identify as a rectangular hyperbola. The equation that describes such a relationship is as follows:

$$y=\frac{a\times x}{x+b}$$

The two constants *a* and *b* thus allow us to describe this hyperbolic relationship, just as with a linear relationship (*y* = *mx* + *c*), which can be expressed by the two constants *m* (the slope) and *c* (the intercept).

We have in fact already defined the constant *a* — it is *V*_max_. The constant *b* is a little more complex, as it is the value on the *x*-axis that gives half of the maximal value of *y*. In enzymology we refer to this as the Michaelis constant (*K*_m_), which is defined as the substrate concentration that gives half-maximal velocity.

Our final equation, usually called the Michaelis–Menten equation, therefore becomes:

$$\text{Initial rate of reaction}(v_{0})=\frac{V_{\max}\times \text{Substrate concentration}}{\text{Substrate concentration}+K_{m}}$$

In 1913, Leonor Michaelis and Maud Menten first showed that it was in fact possible to derive this equation mathematically from first principles, with some simple assumptions about the way in which an enzyme reacts with a substrate to form a product. Central to their derivation is the concept that the reaction takes place via the formation of an ES complex which, once formed, can either dissociate (productively) to release product, or else dissociate in the reverse direction without any formation of product. Thus the reaction can be represented as follows, with *k*_1_, *k*_−1_ and *k*_2_ being the rate constants of the three individual reaction steps:

$$E+S\overset{k_{1}}{\underset{k_{-1}}{⇌}}\mathrm{ES}\overset{k_{2}}{⟶}E+P$$

The Michaelis–Menten derivation requires two important assumptions. The first assumption is that we are considering the initial velocity of the reaction (*v*_0_), when the product concentration will be negligibly small (i.e. [S] ≫ [P]), such that we can ignore the possibility of any product reverting to substrate. The second assumption is that the concentration of substrate greatly exceeds the concentration of enzyme (i.e. [S]≫[E]).

The derivation begins with an equation for the expression of the initial rate, the rate of formation of product, as the rate at which the ES complex dissociates to form product. This is based upon the rate constant *k*_2_ and the concentration of the ES complex, as follows:

1 $$v_{0}=\frac{d[P]}{\mathrm{dt}}=k_{2}\times \left[\mathrm{ES}\right]$$

Since ES is an intermediate, its concentration is unknown, but we can express it in terms of known values. In a steady-state approximation we can assume that although the concentration of substrate and product changes, the concentration of the ES complex itself remains constant. The rate of formation of the ES complex and the rate of its breakdown must therefore balance, where:

$$\text{Rate of ES complex formation}=k_{1}[E][S]$$

and

$$\text{Rate of ES complex breakdown}=(k_{-1}+k_{2})[\mathrm{ES}]$$

Hence, at steady state:

$$k_{1}[E][S]=k_{-1}+k_{2}[\mathrm{ES}]$$

This equation can be rearranged to yield [ES] as follows:

2 $$[\mathrm{ES}]=\frac{k_{1}[E][S]}{k_{-1}+k_{2}}$$

The Michaelis constant *K*_m_ can be defined as follows:

$$K_{m}=\frac{k_{-1}+k_{2}}{k_{1}}$$

Equation 2 may thus be simplified to:

3 $$[\mathrm{ES}]=\frac{\left[E][S\right]}{K_{m}}$$

Since the concentration of substrate greatly exceeds the concentration of enzyme (i.e. [S] ≫ [E]), the concentration of uncombined substrate [S] is almost equal to the total concentration of substrate. The concentration of uncombined enzyme [E] is equal to the total enzyme concentration [E]_T_ minus that combined with substrate [ES]. Introducing these terms to Equation 3 and solving for ES gives us the following:

4 $$[\mathrm{ES}]=\frac{{\left[E\right]}_{T}[S]}{[S]+K_{m}}$$

We can then introduce this term into Equation 1 to give:

5 $$v_{0}=k_{2}[E{]}_{T}\frac{\left[S\right]}{[S]+K_{m}}$$

The term *k*_2_[E]_T_ in fact represents *V*_max_, the maximal velocity. Thus Michaelis and Menten were able to derive their final equation as:

$$v_{0}=\frac{V_{\max}[S]}{[S]+K_{m}}$$

A more detailed derivation of the Michaelis–Menten equation can be found in many biochemistry textbooks (see section 4 of Recommended Reading section). There are also some very helpful web-based tutorials available on the subject.

Michaelis constants have been determined for many commonly used enzymes, and are typically in the lower millimolar range (Table 5).

**Table 5.** Typical range of values of the Michaelis constant.

Enzyme	*K*_m_ (mmol l^−1^)
Carbonic anhydrase	26
Chymotrypsin	15
Ribonuclease	8
Tyrosyl-tRNA synthetase	0.9
Pepsin	0.3

It should be noted that enzymes which catalyse the same reaction, but which are derived from different organisms, can have widely differing *K*_m_ values. Furthermore, an enzyme with multiple substrates can have quite different *K*_m_ values for each substrate.

A low *K*_m_ value indicates that the enzyme requires only a small amount of substrate in order to become saturated. Therefore the maximum velocity is reached at relatively low substrate concentrations. A high *K*_m_ value indicates the need for high substrate concentrations in order to achieve maximum reaction velocity. Thus we generally refer to *K*_m_ as a measure of the affinity of the enzyme for its substrate—in fact it is an inverse measure, where a high *K*_m_ indicates a low affinity, and vice versa.

The *K*_m_ value tells us several important things about a particular enzyme.

1. An enzyme with a low *K*_m_ value relative to the physiological concentration of substrate will probably always be saturated with substrate, and will therefore act at a constant rate, regardless of variations in the concentration of substrate within the physiological range.
2. An enzyme with a high *K*_m_ value relative to the physiological concentration of substrate will not be saturated with substrate, and its activity will therefore vary according to the concentration of substrate, so the rate of formation of product will depend on the availability of substrate.
3. If an enzyme acts on several substrates, the substrate with the lowest *K*_m_ value is frequently assumed to be that enzyme's ‘natural’ substrate, although this may not be true in all cases.
4. If two enzymes (with similar *V*_max_) in different metabolic pathways compete for the same substrate, then if we know the *K*_m_ values for the two enzymes we can predict the relative activity of the two pathways. Essentially the pathway that has the enzyme with the lower *K*_m_ value is likely to be the ‘preferred pathway’, and more substrate will flow through that pathway under most conditions. For example, phosphofructokinase (PFK) is the enzyme that catalyses the first committed step in the glycolytic pathway, which generates energy in the form of ATP for the cell, whereas glucose-1-phosphate uridylyltransferase (GUT) is an enzyme early in the pathway leading to the synthesis of glycogen (an energy storage molecule). Both enzymes use hexose monophosphates as substrates, but the *K*_m_ of PFK for its substrate is lower than that of GUT for its substrate. Thus at lower cellular hexose phosphate concentrations, PFK will be active and GUT will be largely inactive. At higher hexose phosphate concentrations both pathways will be active. This means that the cells only store glycogen in times of plenty, and always give preference to the pathway of ATP production, which is the more essential function.

**Figure 7.**
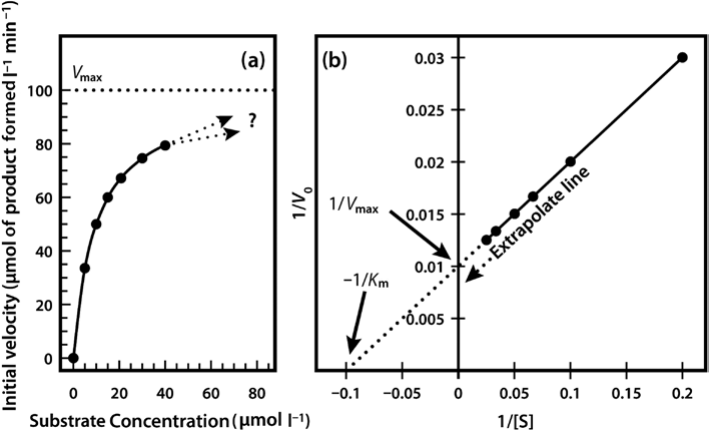
(a) Direct plot. (b) Lineweaver–Burk plot of the same kinetic data.

Very often it is not possible to estimate *K*_m_ values from a direct plot of velocity against substrate concentration (as shown in Figure 6) because we have not used high enough substrate concentrations to come even close to estimating maximal velocity, and therefore we cannot evaluate half-maximal velocity and thus *K*_m_. Fortunately, we can plot our experimental data in a slightly different way in order to obtain these values. The most commonly used alternative is the Lineweaver–Burk plot (often called the double-reciprocal plot). This plot linearizes the hyperbolic curved relationship, and the line produced is easy to extrapolate, allowing evaluation of *V*_max_ and *K*_m_. For example, if we obtained only the first seven data points in Figure 6, we would have difficulty estimating *V*_max_ from a direct plot as shown in Figure 7a.

However, as shown in Figure 7b, if these seven points are plotted on a graph of 1/velocity against 1/substrate concentration (i.e. a double-reciprocal plot), the data are linearized, and the line can be easily extrapolated to the left to provide intercepts on both the *y*-axis and the *x*-axis, from which *V*_max_ and *K*_m_, respectively, can be evaluated.

**Figure 8.**
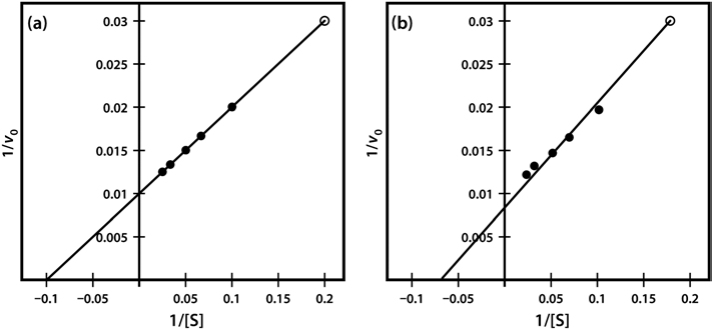
Lineweaver–Burk plot of similar kinetic data, which differ only in a single. (Final data point (a) 1/v 0.03 at 1/S of 0.2 and (b) 1/v 0.031 at 1/S of 0.18).

One significant practical drawback of using the Lineweaver–Burk plot is the excessive influence that it gives to measurements made at the lowest substrate concentrations. These concentrations might well be the most prone to error (due to difficulties in making multiple dilutions), and result in reaction rates that, because they are slow, might also be most prone to measurement error. Often, as shown in Figure 8, such points when transformed on the Lineweaver–Burk plot have a significant impact on the line of best fit estimated from the data, and therefore on the extrapolated values of both *V*_max_ and *K*_m_. The two sets of points shown in Figure 8 are identical except for the single point at the top right, which reflects (because of the plot's double-reciprocal nature) a single point derived from a very low substrate concentration and a low reaction rate. However, this single point can have an enormous impact on the line of best fit and the accompanying estimates of kinetic constants.

In fact there are other kinetic plots that can be used, including the Eadie–Hofstee plot, the Hanes plot and the Eisenthal–Cornish-Bowden plot, which are less prone to such problems. However, the Lineweaver–Burk plot is still the most commonly described kinetic plot in the majority of enzymology textbooks, and thus retains its influence in undergraduate education.

### Enzymes are affected by pH and temperature

Various environmental factors are able to affect the rate of enzyme-catalysed reactions through reversible or irreversible changes in the protein structure. The effects of pH and temperature are generally well understood.

**Figure 9.**
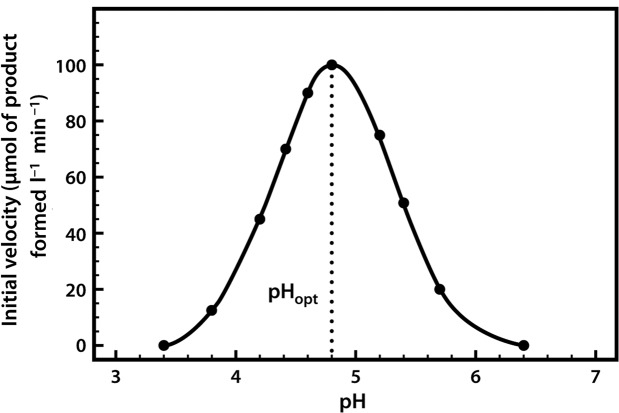
The pH profile of β-glucosidase.

Most enzymes have a characteristic optimum pH at which the velocity of the catalysed reaction is maximal, and above and below which the velocity declines (Figure 9).

The pH profile is dependent on a number of factors. As the pH changes, the ionization of groups both at the enzyme's active site and on the substrate can alter, influencing the rate of binding of the substrate to the active site. These effects are often reversible. For example, if we take an enzyme with an optimal pH (pH_opt_) of 7.0 and place it in an environment at pH 6.0 or 8.0, the charge properties of the enzyme and the substrate may be suboptimal, such that binding and hence the reaction rate are lowered. If we then readjust the pH to 7.0, the optimal charge properties and hence the maximal activity of the enzyme are often restored. However, if we place the enzyme in a more extreme acidic or alkaline environment (e.g. at pH 1 or 14), although these conditions may not actually lead to changes in the very stable covalent structure of the protein (i.e. its configuration), they may well produce changes in the conformation (shape) of the protein such that, when it is returned to pH 7.0, the original conformation and hence the enzyme's full catalytic activity are not restored.

It should be noted that the optimum pH of an enzyme may not be identical to that of its normal intracellular surroundings. This indicates that the local pH can exert a controlling influence on enzyme activity.

**Figure 10.**
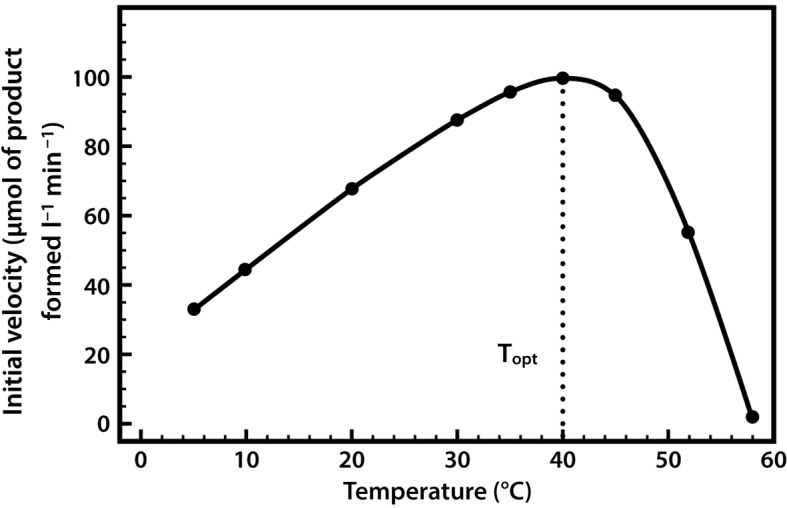
The effect of temperature on enzyme activity.

The effects of temperature on enzyme activity are quite complex, and can be regarded as two forces acting simultaneously but in opposite directions. As the temperature is raised, the rate of molecular movement and hence the rate of reaction increases, but at the same time there is a progressive inactivation caused by denaturation of the enzyme protein. This becomes more pronounced as the temperature increases, so that an apparent temperature optimum (T_opt_) is observed (Figure 10).

Thermal denaturation is time dependent, and for an enzyme the term ‘optimum temperature’ has little real meaning unless the duration of exposure to that temperature is recorded. The thermal stability of an enzyme can be determined by first exposing the protein to a range of temperatures for a fixed period of time, and subsequently measuring its activity at one favourable temperature (e.g. 25°C).

The temperature at which denaturation becomes important varies from one enzyme to another. Normally it is negligible below 30°C, and starts to become appreciable above 40°C. Typically, enzymes derived from microbial sources show much higher thermal stability than do those from mammalian sources, and enzymes derived from extremely thermophilic microorganisms, such as thermolysin (a protease from *Bacillus thermoproteolyticus*) and Taq polymerase (a DNA polymerase from *Thermus aquaticus*), might be completely thermostable at 70°C and still retain substantial levels of activity even at 100°C.

### Enzymes are sensitive to inhibitors

Substances that reduce the activity of an enzyme-catalysed reaction are known as inhibitors. They act by either directly or indirectly influencing the catalytic properties of the active site. Inhibitors can be foreign to the cell or natural components of it. Those in the latter category can represent an important element of the regulation of cell metabolism. Many toxins and also many pharmacologically active agents (both illegal drugs and prescription and over-the-counter medicines) act by inhibiting specific enzyme-catalysed processes.

#### Reversible inhibition

Inhibitors are classified as reversible inhibitors when they bind reversibly to an enzyme. A molecule that is structurally similar to the normal substrate may be able to bind reversibly to the enzyme's active site and therefore act as a competitive inhibitor. For example, malonate is a competitive inhibitor of the enzyme succinate dehydrogenase, as it is capable of binding to the enzyme's active site due to its close structural similarity to the enzyme's natural substrate, succinate (see below). When malonate occupies the active site of succinate dehydrogenase it prevents the natural substrate, succinate, from binding, thereby slowing down the rate of oxidation of succinate to fumarate (i.e. inhibiting the reaction). 
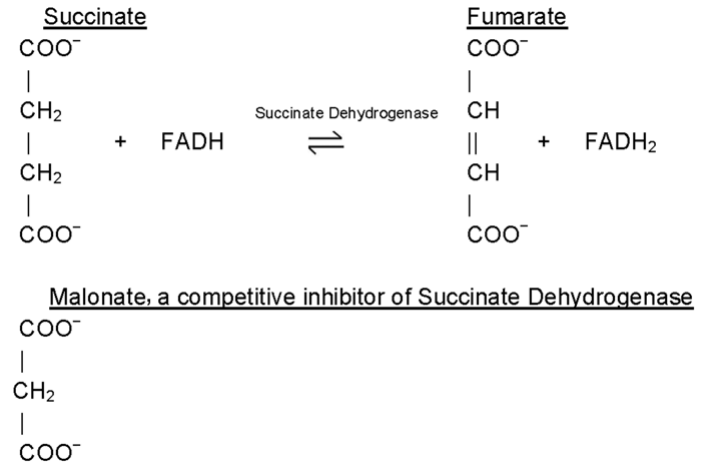


One of the characteristics of competitive inhibitors is that they can be displaced from the active site if high concentrations of substrate are used, thereby restoring enzyme activity. Thus competitive inhibitors increase the *K*_m_ of a reaction because they increase the concentration of substrate required to saturate the enzyme. However, they do not change *V*_max_ itself.

In the case of certain enzymes, high concentrations of either the substrate or the product can be inhibitory. For example, invertase activity is considerably reduced in the presence of high concentrations of sucrose (its substrate), whereas the β-galactosidase of *Aspergillus niger* is strongly inhibited by galactose (its product). Products of an enzyme reaction are some of the most commonly encountered competitive inhibitors.

Other types of reversible inhibitor also exist. Non-competitive inhibitors react with the enzyme at a site distinct from the active site. Therefore the binding of the inhibitor does not physically block the substrate–binding site, but it does prevent subsequent reaction. Most non-competitive inhibitors are chemically unrelated to the substrate, and their inhibition cannot be overcome by increasing the substrate concentration. Such inhibitors in effect reduce the concentration of the active enzyme in solution, thereby reducing the *V*_max_ of the reaction. However, they do not change the value of *K*_m_.

Uncompetitive inhibition is rather rare, occurring when the inhibitor is only able to bind to the enzyme once a substrate molecule has itself bound. As such, inhibition is most significant at high substrate concentrations, and results in a reduction in the *V*_max_ of the reaction. Uncompetitive inhibition also causes a reduction in *K*_m_, which seems somewhat counterintuitive as this means that the affinity of the enzyme for its substrate is actually increased when the inhibitor is present. This effect occurs because the binding of the inhibitor to the ES complex effectively removes ES complex and thereby affects the overall equilibrium of the reaction favouring ES complex formation. It is noteworthy however that since both *V*_max_ and *K*_m_ are reduced the observed reaction rates with inhibitor present are always lower than those in the absence of the uncompetitive inhibitor.

#### Irreversible inhibitors and poisons

If an inhibitor binds permanently to an enzyme it is known as an irreversible inhibitor. Many irreversible inhibitors are therefore potent toxins.

Organophosphorus compounds such as diisopropyl fluorophosphate (DFP) inhibit acetylcholinesterase activity by reacting covalently with an important serine residue found within the active site of the enzyme. The physiological effect of this inactivation is interference with neurotransmitter inactivation at the synapses of nerves, resulting in the constant propagation of nerve impulses, which can lead to death. DFP was originally evaluated by the British as a chemical warfare agent during World War Two, and modified versions of this compound are now widely used as organophosphate pesticides (e.g. parathione, malathione).

### Allosteric regulators and the control of enzyme activity

**Figure 11.**
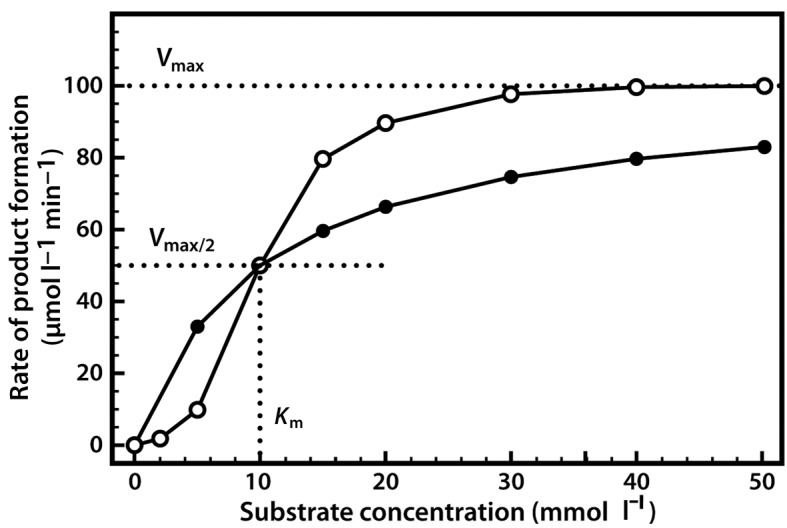
Activity/substrate profiles of allosteric (^) and non-allosteric (•) enzymes with the same affinity and maximal velocity.

Having spent time learning about enzyme kinetics and the Michaelis–Menten relationship, it is often quite disconcerting to find that some of the most important enzymes do not in fact display such properties. Allosteric enzymes are key regulatory enzymes that control the activities of metabolic pathways by responding to inhibitors and activators. These enzymes in fact show a sigmoidal (S-shaped) relationship between reaction rate and substrate concentration (Figure 11), rather than the usual hyperbolic relationship. Thus for allosteric enzymes there is an area where activity is lower than that of an equivalent ‘normal’ enzyme, and also an area where activity is higher than that of an equivalent ‘normal’ enzyme, with a rapid transition between these two phases. This is rather like a switch that can quickly be changed from ‘off’ (low activity) to ‘on’ (full activity).

Most allosteric enzymes are polymeric—that is, they are composed of at least two (and often many more) individual polypeptide chains. They also have multiple active sites where the substrate can bind. Much of our understanding of the function of allosteric enzymes comes from studies of haemoglobin which, although it is not an enzyme, binds oxygen in a similarly co-operative way and thus also demonstrates this sigmoidal relationship. Allosteric enzymes have an initially low affinity for the substrate, but when a single substrate molecule binds, this may break some bonds within the enzyme and thereby change the shape of the protein such that the remaining active sites are able to bind with a higher affinity. Therefore allosteric enzymes are often described as moving from a tensed state or T-state (low affinity) in which no substrate is bound, to a relaxed state or R-state (high affinity) as substrate binds. Other molecules can also bind to allosteric enzymes, at additional regulatory sites (i.e. not at the active site). Molecules that stabilize the protein in its T-state therefore act as allosteric inhibitors, whereas molecules that move the protein to its R-state will act as allosteric activators or promoters.

A good example of an allosteric enzyme is aspartate transcarbamoylase (ATCase), a key regulatory enzyme that catalyses the first committed step in the sequence of reactions that produce the pyrimidine nucleotides which are essential components of DNA and RNA. The reaction is as follows: 
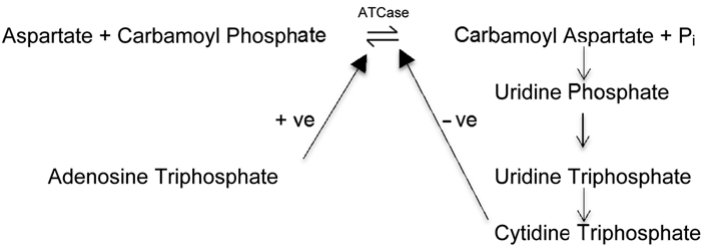


The end product in the pathway, the pyrimidine nucleotide cytidine triphosphate (CTP), is an active allosteric inhibitor of the enzyme ATCase. Therefore when there is a high concentration of CTP in the cell, this feeds back and inhibits the ATCase enzyme, reducing its activity and thus lowering the rate of production of further pyrimidine nucleotides. As the concentration of CTP in the cell decreases then so does the inhibition of ATCase, and the resulting increase in enzyme activity leads to the production of more pyrimidine nucleotides. This negative feedback inhibition is an important element of biochemical homeostasis within the cell. However, in order to synthesize DNA and RNA, the cell requires not only pyrimidine nucleotides but also purine nucleotides, and these are needed in roughly equal proportions. Purine synthesis occurs through a different pathway, but interestingly the final product, the purine nucleotide adenosine triphosphate (ATP), is a potent activator of the enzyme ATCase. This is logical, since when the cell contains high concentrations of purine nucleotides it will require equally high concentrations of pyrimidine nucleotides in order for these two types of nucleotide to combine to form the polymers DNA and RNA. Thus ATCase is able to regulate the production of pyrimidine nucleotides within the cell according to cellular demand, and also to ensure that pyrimidine nucleotide synthesis is synchronized with purine nucleotide synthesis—an elegant biochemical mechanism for the regulation of an extremely important metabolic process.

There are some rare, although important, cases of monomeric enzymes that have only one substrate-binding site but are capable of demonstrating the sigmoidal reaction kinetics characteristic of allosteric enzymes. Particularly noteworthy in this context is the monomeric enzyme glucokinase (also called hexokinase IV), which catalyses the phosphorylation of glucose to glucose-6-phosphate (which may then either be metabolized by the glycolytic pathway or be used in glycogen synthesis). It has been postulated that this kinetic behaviour is a result of individual glucokinase molecules existing in one of two forms—a low-affinity form and a high-affinity form. The low-affinity form of the enzyme reacts with its substrate (glucose), is then turned into the high-affinity form, and remains in that state for a short time before slowly returning to its original low-affinity form (demonstrating a so-called slow transition). Therefore at high substrate concentrations the enzyme is likely to react with a second substrate molecule soon after the first one (i.e. while still in its high-affinity form), whereas at lower substrate concentrations the enzyme may transition back to its low-affinity form before it reacts with subsequent substrate molecules. This results in its characteristic sigmoidal reaction kinetics.

## Origin, purification and uses of enzymes

### Enzymes are ubiquitous

Enzymes are essential components of animals, plants and microorganisms, due to the fact that they catalyse and co-ordinate the complex reactions of cellular metabolism.

Up until the 1970s, most of the commercial application of enzymes involved animal and plant sources. At that time, bulk enzymes were generally only used within the food-processing industry, and enzymes from animals and plants were preferred, as they were considered to be free from the problems of toxicity and contamination that were associated with enzymes of microbial origin. However, as demand grew and as fermentation technology developed, the competitive cost of microbial enzymes was recognized and they became more widely used.

Compared with enzymes from plant and animal sources, microbial enzymes have economic, technical and ethical advantages, which will now be outlined.

### Economic advantages

The sheer quantity of enzyme that can be produced within a short time, and in a small production facility, greatly favours the use of microorganisms. For example, during the production of rennin (a milk-coagulating enzyme used in cheese manufacture) the traditional approach is to use the enzyme extracted from the stomach of a calf (a young cow still feeding on its mother's milk). The average quantity of rennet extracted from a calf's stomach is 10 kg, and it takes several months of intensive farming to produce a calf. In comparison, a 1 000-litre fermenter of recombinant *Bacillus subtilis* can produce 20 kg of enzyme within 12 h. Thus the microbial product is clearly preferable economically, and is free from the ethical issues that surround the use of animals. Indeed, most of the cheese now sold in supermarkets is made from milk coagulated with microbial enzymes (so is suitable for vegetarians).

A further advantage of using microbial enzymes is their ease of extraction. Many of the microbial enzymes used in biotechnological processes are secreted extracellularly, which greatly simplifies their extraction and purification. Microbial intracellular enzymes are also often easier to obtain than the equivalent animal or plant enzymes, as they generally require fewer extraction and purification steps.

Animal and plant sources usually need to be transported to the extraction facility, whereas when microorganisms are used the same facility can generally be employed for production and extraction. In addition, commercially important animal and plant enzymes are often located within only one organ or tissue, so the remaining material is essentially a waste product, disposal of which is required.

Finally, enzymes from plant and animal sources show wide variation in yield, and may only be available at certain times of year, whereas none of these problems are associated with microbial enzymes.

### Technical advantages

Microbial enzymes often have properties that make them more suitable for commercial exploitation. In comparison with enzymes from animal and plant sources, the stability of microbial enzymes is usually high. For example, the high temperature stability of enzymes from thermophilic microorganisms is often useful when the process must operate at high temperatures (e.g. during starch processing).

Microorganisms are also very amenable to genetic modification to produce novel or altered enzymes, using relatively simple methods such as plasmid insertion. The genetic manipulation of animals and plants is technically much more difficult, is more expensive and is still the subject of significant ethical concern, especially in the U.K.

### Enzymes may be intracellular or extracellular

Although many enzymes are retained within the cell, and may be located in specific subcellular compartments, others are released into the surrounding environment. The majority of enzymes in industrial use are extracellular proteins from either fungal sources (e.g. *Aspergillus* species) or bacterial sources (e.g. *Bacillus* species). Examples of these include α-amylase, cellulase, dextranase, proteases and amyloglucosidase. Many other enzymes for non-industrial use are intracellular and are produced in much smaller amounts by the cell. Examples of these include asparaginase, catalase, cholesterol oxidase, glucose oxidase and glucose-6-phosphate dehydrogenase.

### Enzyme purification

Within the cell, enzymes are generally found along with other proteins, nucleic acids, polysaccharides and lipids. The activity of the enzyme in relation to the total protein present (i.e. the specific activity) can be determined and used as a measure of enzyme purity. A variety of methods can be used to remove contaminating material in order to purify the enzyme and increase its specific activity. Enzymes that are used as diagnostic reagents and in clinical therapeutics are normally prepared to a high degree of purity, because great emphasis is placed on the specificity of the reaction that is being catalysed. Clearly the higher the level of purification, the greater the cost of enzyme production. In the case of many bulk industrial enzymes the degree of purification is less important, and such enzymes may often be sold as very crude preparations of culture broth containing the growth medium, organisms (whole or fragmented) and enzymes of interest. However, even when the cheapest bulk enzymes are utilized (e.g. proteases for use in washing powders), the enzyme cost can contribute around 5–10% of the final product value.

#### Pretreatment

At the end of a fermentation in which a microorganism rich in the required enzyme has been cultured, the broth may be cooled rapidly to 5°C to prevent further microbial growth and stabilize the enzyme product. The pH may also be adjusted to optimize enzyme stability. If the enzyme-producing organism is a fungus, this may be removed by centrifugation at low speed. If the enzyme source is bacterial, the bacteria are often flocculated with aluminum sulfate or calcium chloride, which negate the charge on the bacterial membranes, causing them to clump and thus come out of suspension.

#### Treatment

Extracellular enzymes are found in the liquid component of the pretreatment process. However, intracellular enzymes require more extensive treatment. The biomass may be concentrated by centrifugation and washed to remove medium components. The cellular component must then be ruptured to release the enzyme content. This can be done using one or more of the following processes:

• ball milling (using glass beads)
• enzymic removal of the cell wall
• freeze–thaw cycles
• liquid shearing through a small orifice at high pressure (e.g. within a French press)
• osmotic shock
• sonication.

Separation of enzymes from the resulting solution may then involve a variety of separation processes, which are often employed in a sequential fashion.

The first step in an enzyme purification procedure commonly involves separation of the proteins from the non-protein components by a process of salting out. Proteins remain in aqueous solution because of interactions between the hydrophilic (water-loving) amino acids and the surrounding water molecules (the solvent). If the ionic strength of the solvent is increased by adding an agent such as ammonium sulfate, some of the water molecules will interact with the salt ions, thereby decreasing the number of water molecules available to interact with the protein. Under such conditions, when protein molecules cannot interact with the solvent, they interact with each other, coagulating and coming out of solution in the form of a precipitate. This precipitate (containing the enzyme of interest and other proteins) can then be filtered or centrifuged, and separated from the supernatant.

Since different proteins vary in the extent to which they interact with water, it is possible to perform this process using a series of additions of ammonium sulfate, increasing the ionic strength in a stepwise fashion and removing the precipitate at each stage. Thus such fractional precipitation is not only capable of separating protein from non-protein components, but can also enable separation of the enzyme of interest from some of the other protein components.

Subsequently a wide variety of techniques may be used for further purification, and steps involving chromatography are standard practice.

**Ion-exchange chromatography** is often effective during the early stages of the purification process. The protein solution is added to a column containing an insoluble polymer (e.g. cellulose) that has been modified so that its ionic characteristics will determine the type of mobile ion (i.e. cation or anion) it attracts. Proteins whose net charge is opposite to that of the ion-exchange material will bind to it, whereas all other proteins will pass through the column. A subsequent change in pH or the introduction of a salt solution will alter the electrostatic forces, allowing the retained protein to be released into solution again.

**Gel filtration** can be utilized in the later stages of a purification protocol to separate molecules on the basis of molecular size. Columns containing a bed of cross-linked gel particles such as Sephadex are used. These gel particles exclude large protein molecules while allowing the entry of smaller molecules. Separation occurs because the larger protein molecules follow a path down the column between the Sephadex particles (occupying a smaller fraction of the column volume). Larger molecules therefore have a shorter elution time and are recovered first from the gel filtration column.

**Affinity chromatography** procedures can often enable purification protocols to be substantially simplified. Typically, with respect to enzyme purification, a column would be packed with a particulate stationary phase to which a ligand molecule such as a substrate analogue, inhibitor or cofactor of the enzyme of interest would be firmly bound. As the sample mixture is passed through the column, the enzyme interacts with, and binds, to the immobilised ligand, being retained within the column as all of the other components of the mixture pass through the column unrewarded. Subsequently a solution of the ligand is introduced to the column to release (elute) and thereby recover the bound enzyme from the column in a highly purified form.

Nowadays numerous alternative affinity chromatography procedures exist that are able to separate enzymes by binding to areas of the molecule away form their active site. Advances in molecular biology enable us to purify recombinant proteins, including enzymes, through affinity tagging. In a typical approach the gene for the enzyme of interest would be modified to code for a further short amino acid sequence at either the N- or C- terminal. For example, a range of polyhistidine tagging procedures are available to yield protein products with six or more consecutive histidine residues at their N- or C- terminal end. When a mixture containing the tagged protein of interest is subsequently passed through a column containing a nickel-nitrilotriacetic acid (Ni-NTA) agarose resin, the histidine residues on the recombinant protein bind to the nickel ions attached to the support resin, retaining the protein, whilst other protein and non-protein components pass through the column. Elution of the bound protein can then be accomplished by adding imidazole to the column, or by reducing the pH to 5-6 to displace the His-tagged protein from the nickel ions.

Such techniques are therefore capable of rapidly and highly effectively isolating an enzyme from a complex mixture in only one step, and typically provide protein purities of up to 95%. If more highly purified enzyme products are required, other supplemental options are also available, including various forms of preparative electrophoresis e.g. disc-gel electrophoresis and isoelectric focusing.

#### Finishing of enzymes

Enzymes are antigenic, and since problems occurred in the late 1960s when manufacturing workers exhibited severe allergic responses after breathing enzyme dusts, procedures have now been implemented to reduce dust formation. These involve supplying enzymes as liquids wherever possible, or increasing the particle size of dry powders from 10 μm to 200–500 μm by either prilling (mixing the enzyme with polyethylene glycol and preparing small spheres by atomization) or marumerizing (mixing the enzyme with a binder and water, extruding long filaments, converting them into spheres in a marumerizer, drying them and covering them with a waxy coat).

## Industrial enzymology

Although many industrial processes, such as cheese manufacturing, have traditionally used impure enzyme sources, often from animals or plants, the development of much of modern industrial enzymology has gone hand in hand with the commercial exploitation of microbial enzymes. These were introduced to the West in around 1890 when the Japanese scientist Jokichi Takamine settled in the U.S.A. and set up an enzyme factory based on Japanese technology. The principal product was Takadiastase, a mixture of amylolytic and proteolytic enzymes prepared by cultivating the fungus *Aspergillus oryzae* on rice or wheat bran. Takadiastase was marketed successfully in the U.S.A. as a digestive aid for the treatment of dyspepsia, which was then believed to result from the incomplete digestion of starch.

Bacterial enzymes were developed in France by August Boidin and Jean Effront, who in 1913 found that *Bacillus subtilis* produced a heat-stable α-amylase when grown in a liquid medium made by extraction of malt or grain. The enzyme was primarily used within the textile industry for the removal of the starch that protects the warp in the manufacture of cotton.

In around 1930 it was found that fungal pectinases could be used in the preparation of fruit products. In subsequent years, several other hydrolases were developed and sold commercially (e.g. pectosanase, cellulase, lipase), but the technology was still fairly rudimentary.

After World War Two the fermentation industry underwent rapid development as methods for the production of antibiotics were developed. These methods were soon adapted for the production of enzymes. In the 1960s, glucoamylase was introduced as a means of hydrolysing starch, replacing acid hydrolysis. Subsequently, in the 1960s and 1970s, proteases were incorporated into detergents and then glucose isomerase was introduced to produce sweetening agents in the form of high-fructose syrups. Since the 1990s, lipases have been incorporated into washing powders, and a variety of immobilized enzyme processes have been developed (see section on enzyme immobilization), many of which utilize intracellular enzymes.

Currently, enzymes are used in four distinct fields of commerce and technology (Table 6):

• as industrial catalysts
• as therapeutic agents
• as analytic reagents
• as manipulative tools (e.g. in genetics).

**Table 6.** Uses of industrial enzymes.

Enzyme	Reaction	Source	Application
**Industrial catalysts**			
Acid proteases	Protein digestion	*Aspergillus niger*, *Kluyveromyces lactis*	Milk coagulation in cheese manufacture
Alkaline proteases	Protein digestion	*Bacillus* species	Detergents and washing powders
Aminoacylase	Hydrolysis of acylated l–amino acids	*Aspergillus* species	Production of l–amino acids
α-Amylase	Starch hydrolysis	*Bacillus* species	Conversion of starch to glucose or dextrans in the food industry
Amyloglucosidase	Dextrin hydrolysis	*Aspergillus* species	Glucose production
β-Galactosidase	Lactose hydrolysis	*Aspergillus* species	Hydrolysis of lactose in milk or whey
Glucose isomerase	Conversion of glucose to fructose	*Streptomyces* species	High-fructose syrup production
Penicillin acylase	Penicillin side-chain cleavage	*E. coli*	6-APA formation for production of semi-synthetic penicillins
**Therapeutic agents**			
l-Asparaginase	Removal of l–asparagine essential for tumour growth	*E. coli*	Cancer chemotherapy, particularly for leukaemia
Urokinase	Plasminogen activation	Human	Removal of fibrin clots from bloodstream
**Analytic reagents**			
Glucose oxidase	Glucose oxidation	*Aspergillus niger*	Detection of glucose in blood
Luciferase	Bioluminescence	Marine bacteria or firefly	Bioluminescent assays involving ATP
Peroxidase	Dye oxidation using H_2_O_2_	Horseradish	Quantification of hormones and antibodies
Urease	Hydrolysis of urea to CO_2_ and NH_3_	Jack bean	Urea quantification in body fluids
**Manipulative tools**			
Lysozyme	Hydrolysis of 1–4 glycosidic bonds	Hen egg white	Disruption of mucopeptide in bacterial cell walls
Nucleases	Hydrolysis of phosphodiester bonds	Various bacteria	Restriction enzymes used in genetic manipulation to cut DNA
DNA polymerases	DNA synthesis	*Thermus aquaticus*	DNA amplification used in the polymerase chain reaction

Of the thousands of different types of enzymes, about 95% are available from suppliers in quantities ranging from μg to kg, provided essentially for research purposes. Around 40–50 enzymes are produced on an industrial scale (i.e. ranging from multiple kilograms to tonnes per annum). The global enzyme market is currently dominated by the hydrolases, especially the proteases, together with amylases, cellulases and lipases supplied either as liquid concentrates or as powders or granules that release the soluble enzyme on dissolution. Global production is dominated by two companies, which between them supply more than two-thirds of the global enzyme market, namely the Danish company Novozymes, with a market share of 47%, and the U.S. company DuPont (which recently acquired Genencor), with 21%.

The value of the world enzyme market has increased steadily from £110 million in 1960 to £200 million in 1970, £270 million in 1980, £1 000 million in 1990 and over £2 000 million in 2010. Food and beverage enzymes represented the largest sector of the industrial enzymes market in 2010, with a value of £750 million, and the market for enzymes for technical applications (including diagnostic applications, research and biotechnology) accounted for a further £700 million. Estimates of future demand are in the range of £4 000–5 000 million between 2015 and 2016, growing at a rate of 6–7% annually. The developing economies of the Asia-Pacific Region, the Middle East and Africa are now seen to be emerging as the fastest growing markets for industrial enzymes.

Microbial enzymes are typically produced in batches by culturing the producing organism within a batch fermenter. Fermentation typically lasts between 30 and 150 h, with the optimum enzyme yield for the process falling somewhere between the optimum biomass yield and the point of maximal enzyme activity within the cells. Relatively small fermenters with a volume of 10–100 m^3^ are generally employed, allowing flexibility where a number of different products are being produced. Many production systems are optimized by means of a fed-batch process, in which substrates are gradually fed into the reactor over the course of the fermentation, rather than being provided all at once at the start of the process. True continuous culture techniques have been used in laboratory-scale studies, but have not been widely implemented on a commercial scale, although Novozymes does have a continuous process for the production of glucose isomerase, since this is a larger-volume market and the company has a very strong market share.

### Enzyme immobilization

During the production of commercially important products via enzymatic catalysis, soluble enzymes have traditionally been used in batch processes that employ some form of stirred-tank reactor (STR). In these processes, at the end of the batch run the product must be separated from any unused substrate, and also from the enzyme catalyst. Removal of the enzyme at this stage can be achieved by thermal denaturation (only if the product is thermostable) or by ammonium sulfate precipitation or ultrafiltration. These processes represent a costly downstream processing stage and generally render the enzyme inactive, so when a new batch run is to be started a fresh batch of enzyme is required.

**Figure 12.**
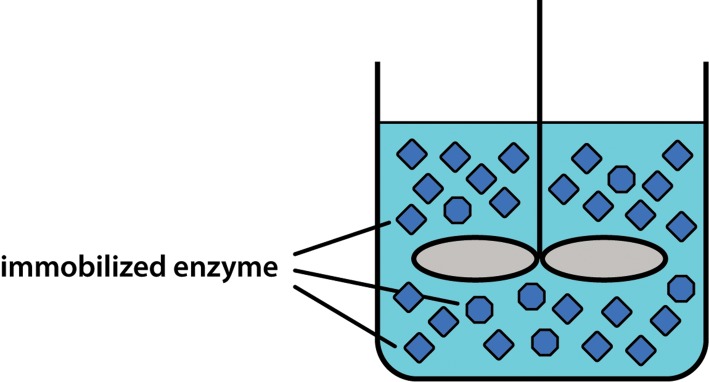
Stirred-tank reactor containing immobilized enzyme.

Immobilized enzyme systems, in contrast, ‘fix’ the enzyme so that it can be reused many times, which has a significant impact on production costs. As a very simple example, if an enzyme is mixed with a solution of warm (but not too hot) agar and this is allowed to set, the enzyme will be entrapped (for the purposes of this example let us ignore the fact that the enzyme will gradually leak out of this gel). The agar can then be cut up into cubes and these can be placed in a STR, together with substrate, as shown in Figure 12. Again the reaction would be allowed to proceed (and it might actually be slower due to diffusional constraints and other effects described later). At the end of the batch run the catalyst can now be easily separated from the product by passing the reactor contents through a coarse mesh. Immediately an important downstream processing step has been carried out and, just as importantly, the active enzyme has been recovered so that it can be reused for the next batch run. This ease of separation of enzyme from product is a major advantage of all immobilized systems over their counterparts that use free (i.e. soluble) enzyme.

This physical advantage of ease of reuse of immobilized biocatalysts is one of the main reasons why such systems are favoured commercially. However, immobilization may also produce biochemical changes that lead to enhanced biocatalyst stability, which may be manifested as:

• an increased rate of catalysis
• prolonged duration of catalysis
• greater operational stability to extremes of pH, temperature, etc.

The particular advantage(s) conferred by immobilization will therefore differ from one system to another. It should be noted that often there may be no biochemical advantage at all, and the simple physical advantage of ease of separation of the biocatalyst from the product may be sufficient to favour the commercial development of an immobilized process.

At this point one problem that will immediately spring to mind for most students is that they have always been taught to fully mix all of the reagents of a reaction, yet the basic principle of immobilization is to partition the biocatalyst into a distinct phase, rather than mix it homogeneously with the substrate. Will this not cause reaction rates to be low? The answer to this question is yes, and the relationship between the activity of an immobilized system and a non-immobilized system can be expressed as the effectiveness factor (η), where:

$$\text{Effectiveness factor}=\frac{\text{Activity of immobilized biocatalyst}}{\text{Activity of non - immobilized biocatalyst}}$$

Thus an immobilized system with an effectiveness factor of 0.1 would show only 10% of the activity of a non-immobilized system with the same amount of enzyme and operating under the same conditions. At first sight this might appear to be a major problem. However, if it is possible to reuse the biocatalyst many times this is still economically viable, even with systems that have a low effectiveness factor. In principle, therefore, for economic viability:

Effectiveness factor×Number of times of reuse≥1

Thus if an immobilized system has an effectiveness factor of 0.1 (i.e. 10%) and we can reuse the biocatalyst 10 times, we essentially achieve the same overall catalytic activity with both the non-immobilized system and the immobilized one. However, if we are able to reuse the biocatalyst 100 times we in fact obtain 10 times more total activity from the immobilized system than from the equivalent non-immobilized system, so the immobilized system may be economically preferable.

**Figure 13.**
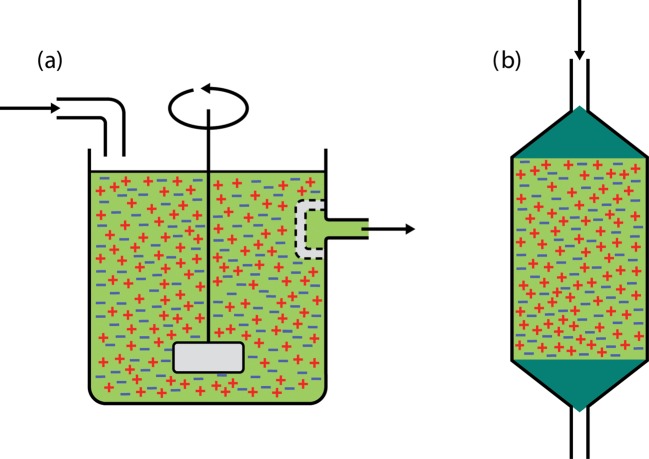
Continuous-flow reactors. (a) Continuous-flow stirred-tank reactor (CSTR). (b) Packed-bed reactor (PBR).

Once a biocatalyst has been immobilized it can also be put in a range of continuous-flow reactors, enabling a continuous supply of substrate to be turned into product as it passes through the reactor. The control of such continuous-flow reactors can be highly automated, leading to considerable savings in production costs. For example, a STR can be easily modified to produce a continuous-flow stirred-tank reactor (CSTR) (Figure 13a), in which the enzyme is held within the reactor by a coarse mesh, and the product continuously flows out of the reactor as substrate is pumped in. It is also possible to produce a packed-bed reactor (PBR) (Figure 13b), in which the agar cubes are packed into a column and the substrate is pumped through the bed without any need for stirring.

CSTRs and PBRs enable the enzyme to be reused many times before it needs to be replaced. For example, in the production of high-fructose syrups, the immobilized glucose isomerase enzyme would typically be used continuously for between 2 and 4 months, and only after this time (when its activity would have dropped to 25% of the original level) would it need to be replaced.

The overall operating costs of continuous-flow reactors are often significantly lower than those of equivalent batch processes. Batch reactors need to be emptied and refilled frequently at regular intervals. Not only is this procedure expensive, but it also means that there are considerable periods of time when such reactors are not productive (so-called ‘downtime’). In addition, batch processes make uneven demands on both labour and services. They may also result in pronounced batch-to-batch variations, as the reaction conditions change with time, and they may be difficult to scale up, due to the changing power requirements for efficient mixing. Due to their higher overall process efficiency, continuous processes using immobilized enzymes may be undertaken in production facilities that are around 10 to 100 times smaller than those required for equivalent batch processes using soluble enzymes. Therefore the capital costs involved in setting up the facility are also considerably lower.

#### Immobilization techniques

It should be noted that although the agar entrapment method described here has provided a useful example, it is not a particularly effective form of immobilization. The high temperature required to prevent the agar from setting may lead to thermal inactivation of the enzyme, and the agar gel itself is very porous and will allow the enzyme to leak out into the surrounding solution.

**Figure 14.**
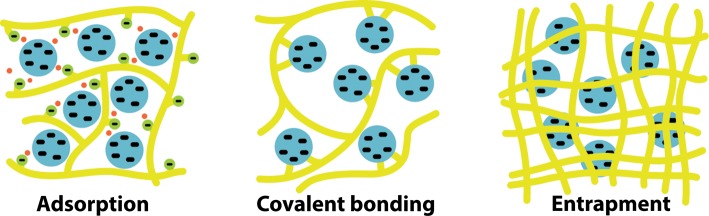
Immobilization techniques.

There are in fact thousands of different techniques of immobilization, all of which are much more effective than our example. In general these techniques can be classified as belonging to one of three categories (Figure 14):

• adsorption
• covalent bonding
• entrapment.

##### Adsorption

The physical adsorption of an enzyme to a supporting matrix is the oldest method of immobilization. As early as 1916, J.M. Nelson and Edward G. Griffin described the adsorption of yeast invertase on to activated charcoal, and the subsequent use of this preparation for sucrose hydrolysis. Over the years a variety of adsorbents have been used, including cellulose, Sephadex, polystyrene, kaolinite, collagen, alumina, silica gel and glass. Such immobilization procedures are extremely easy to perform, as the adsorbent and enzyme are simply stirred together for a time (typically minutes to hours). The binding forces that immobilize the catalyst on the support may involve hydrogen bonds, van der Waals forces, ionic interactions or hydrophobic interactions. Such forces are generally weak in comparison with covalent bonds—for example, a hydrogen bond has an energy content of about 20 kJ mol^−1^, compared with 200–500 kJ mol^−1^ for a covalent bond. Thus, when using such methods, yields (i.e. the amount of enzyme bound per unit of adsorbent) are generally low. In addition, adsorption is generally easily reversed, and can lead to desorption of the enzyme at a critical time.

**Figure 15.**
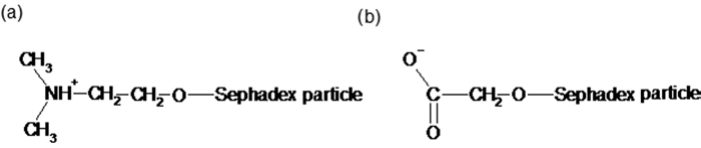
Properties of (a) DEAE–Sephadex and (b) CM–Sephadex ion-exchange resins.

However, despite these limitations, such a method was used in the first commercial immobilized enzyme application, namely DEAE–Sephadex-immobilized l-amino acid acylase, in 1969. DEAE–Sephadex is an ion-exchange resin that consists of an inert dextran particle activated by the addition of numerous diethylaminoethyl groups. Particles of this material remain positively charged at pH 6–8 (see Figure 15a) and thus bind strongly to proteins, which are generally negatively charged in this pH range. If the pH is kept constant, the enzyme and support will remain ionically linked. However, when over time the enzyme loses its activity through denaturation, the pH can be adjusted to a more acidic value, the old enzyme will be desorbed, and the pH can then be readjusted back to pH 6–8 and a fresh batch of enzyme bound. Thus the support matrix may be used many times, giving the process significant economic benefits.

Clearly DEAE–Sephadex immobilization is only of value for enzymes that have a neutral-to-alkaline pH optimum. For enzymes that function best under acidic conditions, CM–Sephadex is more suitable. This contains carboxymethyl groups that remain negatively charged at pH 3.5–4.5 (Figure 15b). Proteins at this pH are generally positively charged and will thus ionically bind to the support. Desorption of the enzyme will occur when the pH is adjusted to a more alkaline value.

Due to the simplicity and controllability of this immobilization procedure, combined with the economic benefits of reuse of the support, ion-exchange materials are now widely used as the method of choice in many industrial settings.

##### Covalent bonding

Immobilization of enzymes by covalent bonding to activated polymers is a widely used approach since, although it is often a tedious procedure, it is capable of producing an immobilized enzyme that is firmly bound to its support. The range of polymers and chemical coupling procedures that are used is enormous.

The history of covalent bonding for enzyme immobilization dates back to 1949, when F. Michael and J. Ewers used the azide derivative of carboxymethylcellulose to immobilize a variety of proteins. Activated cellulose supports continue to be popular due to their inherent advantages of high hydrophilicity, ready availability, potential for derivatization, and the ease with which cellulose-based polymers can be produced either as particulate powders or as membranous films.

It is often more effective not to build the reactive group into the cellulose itself, but instead to use a chemical ‘bridge’ between the cellulose and the enzyme molecule. The requirements for such a bridging or linking molecule are that it must be small, and that once it has reacted with the support it must have a further reactive group capable of reacting with the enzyme. An example of such a bridging molecule is glutaraldehyde, which contains two aldehyde groups, one at either end of its (CH_2_)_3_ moiety. At neutral pH values the aldehyde groups will react with free amino groups. Thus one end of the glutaraldehyde molecule may be attached to the support, and the other to the enzyme.

Covalently immobilized enzymes are strongly bound to their support, so when the proteins denature they are difficult to remove (in contrast to adsorption, as described earlier). Therefore it is usual for both the enzyme and the support to be replaced. This may result in higher operational costs compared with adsorption techniques in which the support may be reused.

##### Entrapment

The entrapment of an enzyme can be achieved in a number of ways:

• inclusion within the matrix of a highly cross-linked polymer
• separation from the bulk phase by a semi-permeable ‘microcapsule’
• dissolution in a distinct non-aqueous phase.

An important feature of entrapment techniques is that the enzyme is not in fact attached to anything. Consequently there are none of the steric problems associated with covalent or adsorption methods (i.e. the possibility of the enzyme binding in such a way that its active site is obstructed by part of the supporting polymer matrix).

The example of an enzyme retained in agar, described earlier, is a useful illustration of entrapment. A preferable alternative involves mixing the catalyst with sodium alginate gel and extruding this into a solution of calcium chloride to produce solid calcium alginate particles. This technique has the advantage of not requiring the use of high temperatures. However, although it is a popular activity in teaching laboratories, outside that setting it is generally unsuitable for the immobilization of purified enzymes, as these are often able to leak out of the gel. Entrapment techniques for purified enzymes are more likely to involve retaining the enzyme behind some form of ultrafiltration membrane. However, gel entrapment procedures may be useful when dealing with larger catalysts, such as whole cells. For example, gel-immobilized living yeast cells have been used successfully in the manufacture of champagne by Moët & Chandon.

#### Immobilization: changes in enzyme properties

Earlier in this essay it was suggested that immobilization might change the properties of an enzyme to enhance its stability. Initially it was believed that such enhanced stability resulted from the formation of bonds between the enzyme and the supporting matrix that physically stabilize the structure of the protein. Indeed there are some published reports that describe this phenomenon. With regard to the stabilization of proteolytic enzymes, which often exhibit more prolonged activity in the immobilized state, this is most probably explained by the fact that such proteases in free solution are prone to autodigestion (i.e. enzyme molecules cleave the peptide bonds of adjacent enzyme molecules), a process that is largely prevented when they are fixed to a supporting matrix.

However, the effects of immobilization are more often due to the supporting matrix changing the microenvironment around the enzyme and/or introducing diffusional constraints that modify the activity of the catalyst. Consider, for example, immobilization of the enzyme by adsorption on to a polyanionic (negatively charged) support such as cellulose. If the substrate is a cation (i.e. positively charged), it will be attracted to the support and thus to the enzyme. In this case the enzyme might well display higher activity, as the substrate concentration in its microenvironment would be higher than that in the surrounding bulk phase. Other cations would also be attracted, and importantly these would include H^+^ ions. Thus the microenvironment would also be enriched in H^+^ ions, so the pH surrounding the enzyme would be lower than the pH of the bulk phase. Consequently the enzyme would also exhibit an altered pH profile compared with that of its soluble counterpart.

In addition, the immobilization matrix might act as a barrier to the diffusion of substrates, products and other molecules. For example, if a high enzyme loading was put into a gel particle and this was then immersed in substrate solution, the substrate would diffuse into the gel and rapidly be converted into product. Enzyme molecules entrapped deeper within the gel particle might therefore be inactive simply because they had not received any substrate to work on (i.e. all of the substrate was converted to product in the outer layers of the particle). Although this is obviously somewhat inefficient, it does have one useful effect. When over time the enzyme within the system denatures, the loss of activity of the enzyme in the outer part of the particle means that substrate will now diffuse deeper into the particle to reach the previously unused core enzyme molecules. In effect this inner reserve of enzyme will offset the loss of enzyme activity through denaturation, so the system will show little or no overall loss of activity. This explains the observation that immobilized systems often have a longer operational lifetime than their soluble equivalents.

In addition, it is of interest that enzymes bound to natural cell membranes (phospholipid bilayers) within living cells will also probably demonstrate these effects, and immobilized systems thus provide useful models for the study of such membrane-bound proteins in living cells.

#### Immobilized enzymes at work

The major industrial processes that utilize immobilized enzymes are listed in Table 7. Sales of immobilized enzymes peaked in 1990, when they accounted for about 20% of all industrial enzyme sales, almost entirely due to the use of glucose isomerase for the production of sweetening agents. Other commercial applications utilize penicillin acylase, fumarase, β–galactosidase and amino acid acylase. Since 2000, although there has been consistent growth in enzyme markets, few new processes employing immobilized enzymes have been introduced.

**Table 7.** The major industrial processes that use immobilized enzymes.

Process	Enzyme	Production rate (ton year^−1^)
High-fructose corn syrup production	Glucose isomerase	10^7^
Acrylamide production	Nitrile hydratase	10^5^
Transesterification of food oils	Lipase	10^5^
Lactose hydrolysis	Lactase	10^5^
Semi-synthetic penicillin production	Penicillin acylase	10^4^
l-aspartic acid production	Aspartase	10^4^
Aspartame production	Thermolysin	10^4^

The following three examples highlight many of the biochemical, technological and economic considerations relating to the use of immobilized enzymes on a commercial and industrial scale.

##### Production of high-fructose syrup

Undoubtedly the most significant large-scale application of immobilized enzymes involves the production of high-fructose corn syrup (HFCS). Although most of the general public believe that sucrose is responsible for the ‘sweetness’ of food and drinks, there have been significant efforts to replace sucrose with alternative, and often cheaper, soluble caloric sweetening agents. HFCS is a soluble sweetener that has been used in many carbonated soft drinks since the 1980s, including brand-name colas such as Coca-Cola and Pepsi-Cola. HFCS is produced by the enzymatic digestion of starch derived from corn (maize). Developments in HFCS production have been most prominent in countries such as the U.S.A., which have a high capacity to produce starch in the form of corn, but which do not cultivate significant amounts of sugar cane or sugar beet, and must therefore import either the raw products (for processing) or the refined sugar (sucrose) itself.

Simple corn syrups can be manufactured by breaking down starch derived from corn using the enzyme glucoamylase alone or in combination with α-amylase. These enzymes are cheap and can be used in a soluble form. Since starch has to be extracted from corn at high temperatures (because starch has poor solubility at low temperatures and forms very viscous solutions), the process utilizes enzymes from thermophilic organisms, which have very high temperature optima. Simple corn syrup is therefore composed predominantly of glucose, which unfortunately has only 75% of the sweetness of sucrose. However, in order to make the syrup sweeter the enzyme glucose isomerase, which catalyses the following reaction, can be employed:

$$\mathrm{Glucose}\overset{\text{Glucose isomerase}}{⇌}\mathrm{Fructose}$$

This enzyme (described previously in the section on properties and mechanisms of enzyme action) will produce a roughly 50:50 mixture of glucose and fructose at equilibrium, and since fructose has 150% of the sweetness of sucrose, this glucose:fructose mixture will have a similar level of sweetness to sucrose. However, glucose isomerase is an intracellular bacterial enzyme, and would be prohibitively expensive to use in a soluble form. This makes it an ideal candidate for use in an immobilized process.

The first glucose isomerase enzyme to be isolated was obtained from species of *Pseudomona*s in 1957, and more useful enzymes were isolated throughout the 1960s from species of *Bacillus* and *Streptomyces.* In 1967, the Clinton Corn Processing Company of Iowa, U.S.A. (later renamed CPC International) introduced a batch process that utilized an immobilized glucose isomerase enzyme, and by 1972 the company had developed a continuous process for the manufacture of HFCS containing 42% fructose using a glucose isomerase enzyme immobilized on a DEAE ion-exchange support.

During the late 1970s, advances in enzymology, process engineering and fractionation technology led to the production of syrups with a higher fructose content, and today HFCS containing 55% fructose is generally produced, and is commonly used in soft drinks, although 42% fructose syrups are still also produced for use in some processes, including the production of bakery foodstuffs.

In 2010, the U.S. production of HFCS was approximately 8 million metric tons, accounting for 37% of the U.S. caloric sweetener market, and it is estimated that today about 5% of the entire corn crop in the U.S.A. is used to produce HFCS.

##### Hydrolysis of lactose

Within the dairy industry the production of 1 kg of cheese requires about 10 litres of milk, and produces about 9 litres of whey as a waste product. Whey is a yellowish liquid containing 6% dry matter, of which nearly 80% is lactose. The enzyme lactase (β-galactosidase) may be used to break down lactose to its constituent monosaccharides, namely glucose and galactose, which are more soluble than lactose, and have potential uses as carbon sources in microbial fermentation, and can also be used as caloric sweeteners.

$$\mathrm{Lactose}\overset{\mathrm{Lactase}}{⇌}\mathrm{Glucose}+\mathrm{Galactose}$$

Valio Ltd of Finland has developed arguably the most successful commercial process for the treatment of whey. Using a lactase enzyme obtained from *Aspergillus*, immobilized by adsorption and cross-linked on to a support resin, whey syrups are produced that have been utilized as an ingredient in drinks, ice cream and confectionery products. The *Aspergillus* enzyme has an acid pH optimum of 3–5, and by operating at low pH the process avoids excessive microbial contamination. Treatment plants that utilize 600-litre columns have been built in Finland, and these are used to treat 80 000 litres of whey per day. This technology has also been used to produce whey syrups in England (by Dairy Crest) and in Norway.

Similar technology can also be used to remove lactose from milk. Lactose-free milk is produced for consumption by those who have lactose intolerance (a genetic condition), and also for consumption by pets such as cats, which are often unable to digest lactose easily. The first industrial processing facility to use immobilized lactase to treat milk was opened in 1975, when Centrale del Latte of Milan, Italy, utilized a batch process in which yeast (*Saccharomyces*) lactase, with a neutral pH optimum of 6–8, was immobilized within hollow permeable fibres. This process was capable of treating 10 000 litres of milk per day, and was operated at low temperature to prevent microbial contamination.

##### Production of semi-synthetic penicillins

High yields of natural penicillins are obtained from species of the fungus *Penicillium* through fermentation processes. However, over the years many microbial pathogens have become resistant to natural penicillins, and are now only treatable with semi-synthetic derivatives. These are produced through cleavage of natural penicillin, such that the G or V side chain is removed from the 6-aminopenicillanic acid (6-APA) nucleus of the molecule:

$$\text{Penicillin G or V}\overset{\text{Penicillin acylase}}{⇌}\text{G or V side chain}+\text{6 - APA}$$

Thereafter, by attachment of a chemically different side chain, a semi-synthetic penicillin product (e.g. ampicillin, amoxicillin) can be formed. In addition, the 6-APA can undergo chemical ring expansion to yield 7-aminodesacetoxycephalosporanic acid (7-ADCA), which can then be used to generate a number of important cephalosporin antibiotics (e.g. cephalexin, cephradine, cefadroxil).

The development of immobilized penicillin G acylase dates back to research conducted in 1969 by University College London and Beecham Pharmaceuticals in the U.K. Penicillin G acylases are intracellular enzymes found in *E. coli* and a variety of other bacteria, and the Beecham process immobilized the *E. coli* enzyme on a DEAE ion-exchange support. Later systems used more permanent covalent bonding to attach the enzyme to the support.

In the 1980s and 1990s, world production of penicillins was dominated by European manufacturers, which accounted for production of around 30 000 tonnes of penicillin per annum, 75% of which was used for the manufacture of semi-synthetic penicillins and cephalosporins. However, over the past 10 years, due to increasing costs of labour, energy and raw materials, more bulk manufacturing has moved to the Far East, where China, Korea and India have become major producers. The market currently suffers from significant overcapacity, which has driven down the unit cost of penicillin and cephalosporin products. However, penicillins and cephalosporins still represent one of the world's major biotechnology markets, with annual sales of about £10 000 million, accounting for 65% of the entire global antibiotics market.

## Enzymes in analysis

Enzymes have a wide variety of uses in analytical procedures. Their specificity and potency allow both detection and amplification of a target analyte. ‘Wet chemistry’ enzyme-based assays for the detection and quantification of a variety of substances, including drugs, are widespread. Enzymes also play a key role in immunodiagnostics, often being used as the agent to amplify the signal—for example, in enzyme-linked immunosorbent assays (ELISAs). Within DNA-fingerprinting technology, the enzyme DNA polymerase plays a key role in the amplification of DNA molecules in the polymerase chain reaction. However, ‘wet chemistry’ analytical methods are increasingly being replaced by the use of biosensors—that is, self-contained integrated devices which incorporate a biological recognition component (usually an immobilized enzyme) and an electrochemical detector (known as a transducer).

### Biosensors

Much of the technological development of biosensors has been motivated by the need to measure blood glucose levels. In 2000, the World Health Organization estimated that over 170 million people had diabetes, and predicted that this figure will rise to over 360 million by 2030. In view of this, many companies have made significant investments in R&D programmes that have led to the availability of a wide variety of glucose biosensor devices.

In 1962, Leland Clark Jr coined the term ‘enzyme electrode’ to describe a device in which a traditional electrode could be modified to respond to other materials by the inclusion of a nearby enzyme layer. Clark's ideas became a commercial reality in 1975 with the successful launch of the Yellow Springs Instruments (YSI) model 23A glucose analyser. This device incorporated glucose oxidase together with a peroxide-sensitive electrode to measure the hydrogen peroxide (H_2_O_2_) produced during the following reaction:

$$\mathrm{Glucose}+O_{2}\to \text{Gluconic acid}+H_{2}O_{2}$$

In this device, the rate of H_2_O_2_ formation is a measure of the rate of the reaction, which depends on the concentration of glucose in solution, thus allowing the latter to be estimated.

As was discussed earlier, in enzyme-catalysed reactions the relationship between substrate concentration and reaction rate is not linear, but hyperbolic (as described by the Michaelis–Menten equation). This is also true for the glucose oxidase within a biosensor. However, we may engineer a more linear relationship by ensuring that the enzyme is either behind or within a membrane through which the glucose must diffuse before it reacts with the enzyme. This means that the system becomes diffusionally, rather than kinetically, limited, and the response is then more linearly related to the concentration of glucose in solution.

**Figure 16.**
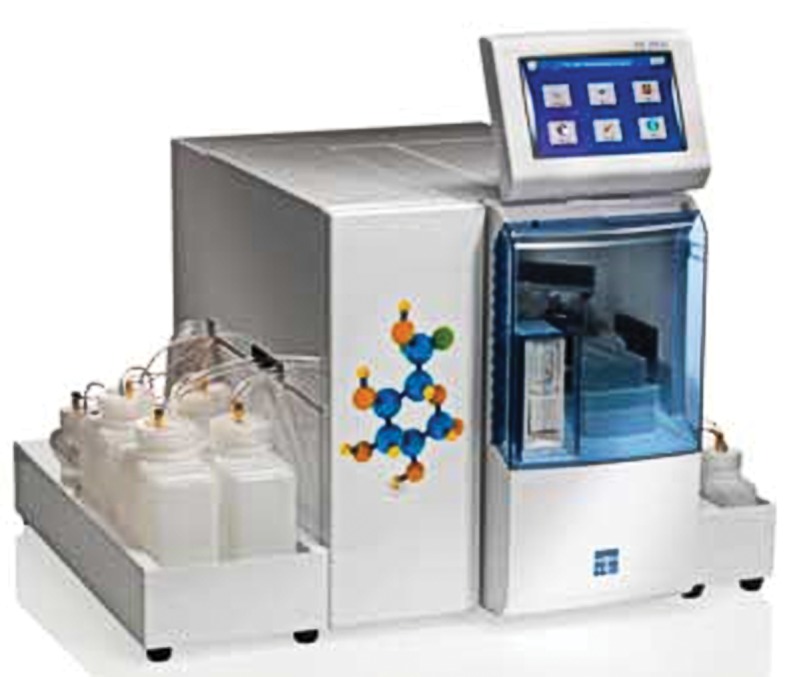
A laboratory-scale glucose analyser. Photograph supplied courtesy of YSI (UK) Limited.

Over the years the YSI model 23A glucose analyser has been replaced by a range of much more advanced models. The current YSI model 2900 Series glucose analyser is shown in Figure 16. This instrument has a 96-sample rack that enables batches of samples to be run, with the analysis of each sample taking less than a minute. The instrument can measure the glucose content of whole blood, plasma or serum, and requires only 10 μl of sample per analysis. The membrane-bound glucose oxidase typically only needs to be replaced every 3 weeks, thereby reducing the cost of analysis. These systems also offer advanced data-handling and data-storage facilities.

In addition, these instruments can be modified to analyse a wide variety of other substances of biological interest, simply by incorporating other oxidase enzymes into the membrane (Table 8).

**Table 8.** Composition of enzyme membranes available for analysers with a peroxide-sensitive electrode as the transducer.

Analyte	Enzyme	Reaction
Glucose	Glucose oxidase	β-D-glucose + O_2_ → gluconic acid + H_2_O_2_
Alcohol	Alcohol oxidase	Ethanol + O_2_ → acetaldehyde + H_2_O_2_
Lactic acid	Lactate oxidase	l-lactate + O_2_ → pyruvate + H_2_O_2_
Lactose	Galactose oxidase	Lactose + O_2_ → galactose dialdehyde derivative + H_2_O_2_

To enable diabetic patients to take their own blood glucose measurements, small hand-held biosensors have also been developed, which are in fact technologically more advanced because the enzyme and transducer are more intimately linked on the sensor surface. The first device of this type was launched in 1986 by Medisense, and was based on technology developed in the U.K. at Cranfield and Oxford Universities. The ExacTech blood glucose meter was the size and shape of a pen, and used disposable electrode strips. This device was followed by a credit card-style meter in 1989. Such devices again rely on glucose oxidase as the biological component, but do not measure the reaction rate via the production (and detection) of H_2_O_2_. Instead they rely on direct measurement of the rate of electron flow from glucose to the electrode surface. The reactions that occur within this device may be summarized as follows:

$$\begin{array}{rl} & \mathrm{Glucose}+GO_{X}-\mathrm{FAD}\to \text{Gluconic acid}+GO_{X}-\mathrm{FAD}H_{2}\\ & GO_{X}-\mathrm{FAD}H_{2}+\text{Ferrocene mediato}r_{oxidized}\to GO_{X}-\mathrm{FAD}+\text{Ferrocene mediato}r_{reduced} \end{array}$$

and at the electrode surface:

$$\text{Ferrocene mediato}r_{reduced}\to \text{Ferrocene mediato}r_{oxidized}+e^{-}$$

where GO_x_-FAD represents the FAD redox centre of glucose oxidase in its oxidized form, and GO_x_-FADH_2_ represents the reduced form.

Basically electrons are removed from the glucose molecules and passed via the enzyme to the ferrocene mediator, which then donates them to the working electrode surface, resulting in the generation of an electrical current that is directly proportional to the rate of oxidation of glucose, and thus proportional to the glucose concentration in the sample.

Medisense, whose only product was its blood glucose meter, was bought by Abbott Diagnostics in 1996, and Abbott-branded devices continued to use and develop this technology for some time.

In 1999, Therasense marketed a glucose meter that represented the next generation of sensing technology, and integrated the enzyme even more closely with the electrode. Originally developed by Adam Heller at the University of Texas in the 1990s, wired-enzyme electrodes do not rely on a soluble mediator such as the ferrocene used in the Medisense devices. Instead the enzyme is immobilized in an osmium-based polyvinyl imidazole hydrogel in which the electrons are passed from enzyme to electrode by a series of fixed electroactive osmium centres that shuttle the electrons onward in a process called ‘electron hopping.’

**Figure 17.**
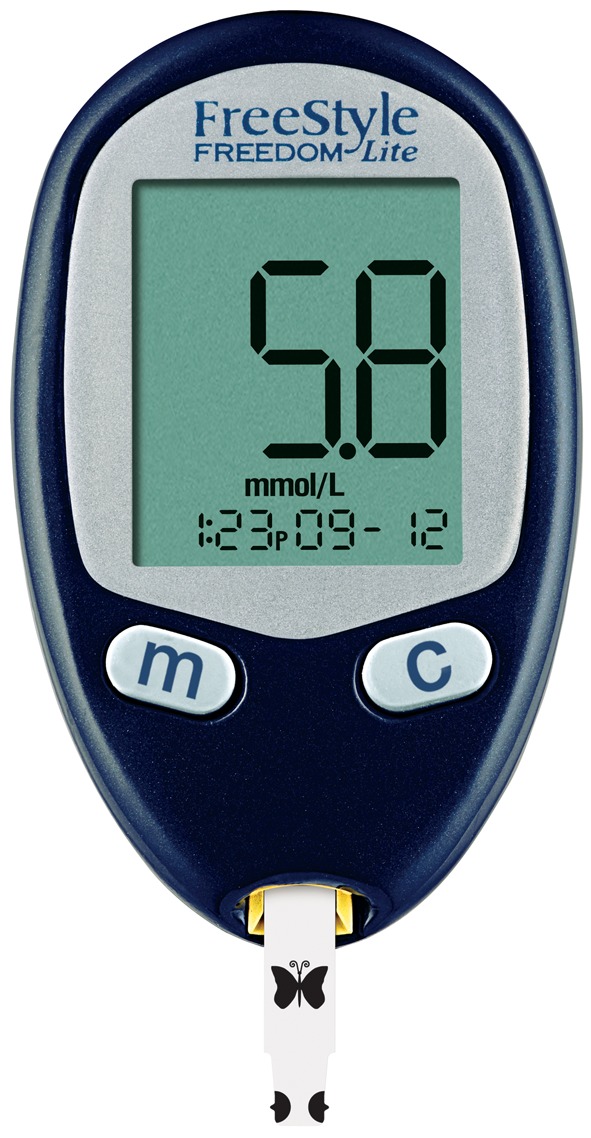
A hand-held glucose biosensor suitable for personal use. Photograph supplied courtesy of Abbott Diabetes Care.

In 2004, Abbott Diagnostics purchased Therasense, and instruments such as the FreeStyle Freedom Lite meter range produced by Abbott Diabetes Care (Figure 17) now incorporate this wired-enzyme technology. Devices of this type are highly amenable to miniaturization.

Continuous measuring devices are becoming increasingly available, and may well revolutionize the control of certain disease conditions. For example, with regard to diabetes, devices such as the FreeStyle Navigator range from Abbott Diabetes Care use the same wired-enzyme technology as that described earlier, but now incorporate this into a tiny filament about the diameter of a thin hypodermic needle. This is inserted approximately 5 mm under the skin to measure the glucose level in the interstitial fluid that flows between the cells. The unit is designed to remain *in situ* for up to 5 days, during which time it can measure the glucose concentration every minute. A wireless transmitter sends the glucose readings to a separate receiver anywhere within a 30-metre range, and this can then issue an early warning alarm to alert the user to a falling or rising glucose level in time for them to take appropriate action and avoid a hypoglycaemic or hyperglycaemic episode.

In addition, experimental units have already been developed that link continuous glucose biosensor measurement systems with pumps capable of gradually dispensing insulin such that the diabetic condition is automatically and reliably controlled, thereby avoiding the traditional peaks and troughs in glucose levels that occur with conventional glucose measurement and the intermittent administration of insulin.

Therefore, looking to the future, we may confidently expect to see the development of biosensor systems that can continuously monitor a range of physiologically important analytes and automatically dispense the required medication to alleviate the symptoms of a number of long-term chronic human illnesses.

## Closing remarks

For the sake of conciseness, this guide has been limited to some of the basic principles of enzymology, together with an overview of the biotechnological applications of enzymes. It is important to understand the relationship between proteins and the nucleic acids (DNA and RNA) that provide the blueprint for the assembly of proteins within the cell. Genetic engineering is thus predominantly concerned with modifying the proteins that a cell contains, and genetic defects (in medicine) generally relate to the abnormalities that occur in the proteins within cells. Much of the molecular age of biochemistry is therefore very much focused on the study of the cell, its enzymes and other proteins, and their functions.

**Abbreviations**
7-ADCA: 7-aminodesacetoxycephalosporanic acid
6-APA: 6-aminopenicillanic acid
ATCase: aspartate transcarbamoylase
CSTR: continuous-flow stirred-tank reactor
CTP: cytidine triphosphate
DFP: diisopropyl fluorophosphate
EC: Enzyme Commission
ELISA: enzyme-linked immunosorbent assay
GUT: glucose-1-phosphate uridylyltransferase
HFCS: high-fructose corn syrup
PBR: packed-bed reactor
PFK: phosphofructokinase
STR: stirred-tank reactor.
